# Supplementary material for: Effectiveness and risk of ARB and ACEi among different ethnic groups in England: A reference trial (ONTARGET) emulation analysis using UK Clinical Practice Research Datalink Aurum-linked data
Source: PLoS Med. 2024 Sep 16;21(9):e1004465. doi: 10.1371/journal.pmed.1004465 (PMC11441682; doi:10.1371/journal.pmed.1004465)
Supplement: S1 Appendix — Table A. Deviations from protocol. Table B. List of variables considered and included in propensity score model for balancing characteristics between exposure groups. Table C. Baseline characteristics and standardised differences of trial-eligible patients after applying trial criteria included in inverse-probability—weighted analysis before and after weighting for the reference trial emulation. Table D. Baseline characteristics and standardised differences of trial-eligible Black patients after applying trial criteria included in inverse-probability—weighted analysis before and after weighting for extending analysis to underrepresented groups. Table E. Baseline characteristics and standardised differences of trial-eligible South Asian patients after applying trial criteria included in inverse-probability—weighted analysis before and after weighting for extending analysis to underrepresented groups. Table F. Baseline characteristics and standardised differences of trial-eligible White patients after applying trial criteria included in inverse-probability—weighted analysis before and after weighting for extending analysis to underrepresented groups. Table G. Number of events for the primary outcome, its components, main secondary outcome, and death from any cause for ARB vs. ACEi using an inverse-probability—weighted analysis of trial-eligible patients in CPRD Aurum after emulation and benchmarking findings against the reference trial (ONTARGET). Table H. Comparative effectiveness of ARB vs. ACEi for the primary and secondary outcomes overall and by ethnicity with a test for multiplicative heterogeneity using an inverse-probability—weighted analysis of trial-eligible patients in CPRD Aurum with an on-treatment approach. Table I. Comparative effectiveness of ARB vs. ACEi for the primary and secondary outcomes overall and by ethnicity using an inverse-probability—weighted analysis of trial-eligible patients in CPRD Aurum with after multiple imputation of missing values [file pmed.1004465.s002.pdf]

Supplementary Material

Development of Propensity Score Model

The propensity score model was developed using a logistic regression model for the probability of being treated with an ACEi for the trial-eligible population.

Covariates

Variables considered as confounders and included in the propensity score model were chosen based on *a-priori* knowledge and the use of a directed-acyclic graph. These included demographics, medication and clinical history, and time-related variables to account for bias introduced in treatment switchers. Categorical variables which had <10% of events were excluded from the initial model.

Time-related variables including time since first eligible period, calendar year of eligible period, number of previous ARB and ACEi periods were included to account for bias introduced from including prevalent users.

Determining functional form of continuous variables

The functional form of continuous variables to meet the linearity assumption was checked using locally weighted scatterplot smoothing. Transformations were then considered for variables which did not satisfy the assumption, until linearity was achieved. Where it was reasonable, categorical forms of the variables were also considered and compared against continuous forms using a likelihood ratio test.

Higher order terms and interactions

Once the functional form of continuous variables had been determined, higher order terms were considered in the model using a likelihood ratio test. If balance was unachieved, interactions between variables were considered in a backwards selection approach.

Checking of assumptions

Once the model was complete, the positivity assumption was checked by assessing the distribution of the propensity scores. If violations occurred and to avoid extreme weights, propensity scores were trimmed at the 1st percentile in the ACEi group and the 99th percentile in the ARB group.

Weighting

Inverse-probability weighting was used to obtain balance of baseline characteristics within treatment groups.

Note on variables with missing data

Variables with missing data >10% were included in the model with a missing indicator if they could be assumed to be missing at random. Variables with large missingness which could not be assumed to missing at random assumption were omitted from the initial model. If balance was unachieved after omitting these variables they would then be considered in the model with a missing indicator.

Due to the importance of blood pressure in this therapeutic area and as these medications are indicated for hypertension in this population, complete records only were unlikely to bias results. Therefore, despite this variable having missing data >10%, blood pressure was included without a missing indicator. The impact of this bias was then assessed in a sensitivity analysis using multiple imputation. Creatinine was included with a missing indicator and the mean imputed for missing values imputed. Alcohol status was omitted due to large missingness and not meeting the missing at random assumption.

Table A. Deviations from protocol	
**Deviation**	**Reason**
Use of Aurum data as opposed to GOLD/Aurum and GOLD combined	GOLD alone did not provide sufficient numbers for the ethnic groups of interest. Aurum alone provided sufficient sample size to study the objectives and due to time contraints of the project it was not necessary to additionally include data from GOLD. Therefore to assess effects among the underrepresented subgroup of Black and South Asian ethnicity, analysis was conducted in Aurum only.
Using propensity-score—weighting as opposed to propensity-score—matching	To obtain average treatment effect as opposed to average treatment effect on treated and increase sample size.
Underrepresented group analysis on propensity-score—weighted sample as opposed to propensity-score—matched cohort	This was to increase sample size as previous work (referenced in the manuscript) showed analyses using both propensity-score—weighting and propensity-score—matching gave almost identical results
Omitting step of matching to individual trial participants when benchmarking	Previous work showed omitting trial matching when benchmarking gained equivalent results. Since the aim of this analysis was to extend to trial underrepresented groups benchmarking was conducted on the trial-eligible cohort which was used throughout
Primary outcome: including both fatal and non-fatal events for stroke and myocardial infarction	Consistency with trial
Included additional outcome- main secondary outcome: composite of cardiovascular-related death, myocardial infarction, or stroke	Consistency with trial
Angina inclusion criteria: Removed condition that needed to have previous coronary artery disease diagnosis	Misclassification

CABG Inclusion criteria: Removed condition that could be with angina	Only included events where CABG was within 4 years prior to avoid due to potential of capturing old events
On-treatment (per-protocol) analysis for secondary objectives 1 and 2 (extending findings to trial-underrepresented and excluded groups)	Not deemed necessary as on-treatment analysis was sufficiently comparable to ITT for primary outcome
Previously mentioned that patients had to meet inclusion and exclusion criteria prior to start of first exposed period instead trial criteria assessed at start of all exposed periods	Incorrect wording in protocol this reduces bias by assessing at start of follow up
Referred to analysis group as trial-analogous now analysis groups will be labelled as propensity-score—weighted trial-eligible for main analysis and propensity-score—matched trial-eligible for sensitivity analysis	To avoid confusion as only the ACEi trial-eligible cohort is trial-matched
Naming of nephropathy outcomes	Changed from nephropathy 1 and nephropathy 2 to loss of eGFR or ESKD and ESKD

Table B List of variables considered and included in propensity-score model for balancing characteristics between exposure groups

**Potential confounders**	**Selected into propensity-score model**	**Missing data**	**Reason for omitting from PS model**
Stroke/TIA	✓	-	
Peripheral artery disease	✓	-	
Coronary artery disease	✓	-	
Diabetes	✓	-	
High-risk diabetes	✓	-	
Age (years)	✓	-	
Sex	✓	-	
Ethnicity	✓	-	
BMI	✓	(58933) 7%	
SBP	✓	(186684) 22%	
DBP	✓	(186701) 22%	
Creatinine	✓	(156849) 19%	
Index of Multiple Deprivation (IMD)	✓	(1157) 0.1%	
Smoke status	✓	(20329) 2.4%	
Alcohol use		(136544) 16%	Missing data
Statin use	✓	-	
Nitrate use	✓	-	
Diabetic treatment use	✓	-	
Diuretic use	✓	-	
CCB use	✓	-	
Betablocker use	✓	-	
Aspirin use	✓	-	
Antiplatelet use	✓	-	
Digoxin use		-	Insufficient number of events
Anticoagulant use	✓	-	
Alpha-blocker use	✓	-	
No. of hospital admissions within 6 months prior	✓	-	
No. of GP appointments within 6 months prior	✓	-	
Year of start of eligible period	✓	-	

Table B List of variables considered and included in propensity-score model for balancing characteristics between exposure groups

**Potential confounders**	**Selected into propensity-score model**	**Missing data**	**Reason for omitting from PS model**
Time since first eligible period (days)	✓	-	
No. of previous ACE inhibitor eligible periods	✓	-	
No. of previous ARB eligible periods	✓	-	

Notes: TIA: transient ischaemic attack; BMI: body-mass index; SBP: systolic blood pressure; DBP: diastolic blood pressure.

Variables are measured at start of trial-eligible period or before.

Peripheral artery disease includes limb bypass surgery or angioplasty, limb/foot amputation, or intermittent claudication.

Coronary artery disease includes previous MI, angina, coronary angioplasty, or CABG.

SBP and DBP are measured within 6 months prior to start of trial-eligible period.

Medication use is within 3 months prior to start of trial-eligible period.

Quantitative variables were included as continuous terms. BMI was included as a categorical variable grouped at: <25, 25-30, 30+ kg/m^2.

SBP and DBP had 22% missing data but this variable was included as believed to be an important confounder and can be assumed to be MAR. Creatinine was included as a binary indicator for missing and non-missing and an additional variable where missing values were imputed as the mean. Alcohol was omitted due to missing data >10% and reason to not assume to be MAR. If balance was unachieved for this variable after inverse-probability—weighting it would be considered in the model with a missing value category.

Balance after weighting was assessed for all variables listed including those not included in the propensity-score model.

Table C Baseline characteristics and standardised differences of trial-eligible patients after applying trial criteria included in inverse-probability—weighted analysis before and after weighting for the reference trial emulation

Characteristic	Before weighting		After weighting		
	ARB N=151,807	ACEi N=421,214	ARB N=554,609	ACEi N=562,686	SMD
**Age (year) – mean (SD)**	71.3 (9.2)	70.9 (9.4)	554609 (71.1)	562686 (71.0)	0.007
**Systolic BP (mmHg) – mean (SD)**	144.0 (20.2)	143.6 (20.2)	144.7 (20.6)	144.1 (20.3)	0.031
**Diastolic BP (mmHg) – mean (SD)**	78.8 (10.9)	79.1 (11.0)	79.3 (11.0)	79.2 (11.0)	0.015
**Body-mass index – mean (SD)**	29.2 (5.8)	28.8 (5.8)	28.9 (5.7)	28.9 (5.8)	0.010
**Creatinine (µmol/l) – mean (SD)**	94.1 (29.5)	92.9 (27.3)	93.8 (28.6)	93.5 (28.1)	0.012
**Female sex – no. (%)**	82849 (54.6)	202511 (48.1)	278912 (50.3)	280169 (49.8)	0.010
**Ethnic group – no. (%)**					
Black	5947 (3.9)	11646 (2.8)	17144 (3.1)	17371 (3.1)	0.000
South Asian	10668 (7.0)	20137 (4.8)	30176 (5.4)	30309 (5.4)	0.002
White	135192 (89.1)	389431 (92.5)	507289 (91.5)	515006 (91.5)	0.002
**Clinical history – no. (%)**					
CAD[a]	105969 (69.8)	298697 (70.9)	389536 (70.2)	396723 (70.5)	0.006
Cerebrovascular disease[b]	16071 (10.6)	44821 (10.6)	58605 (10.6)	59511 (10.6)	0.000
PAD[c]	14158 (9.3)	38958 (9.3)	51159 (9.2)	52018 (9.2)	0.001
Diabetes	93709 (61.7)	250030 (59.4)	333282 (60.1)	337295 (59.9)	0.003
High-risk diabetes[d]	76297 (50.3)	197471 (46.9)	270032 (48.7)	270292 (48.0)	0.013
**Smoking status – no. (%)**					
Non-smoker	44137 (29.1)	113688 (27.0)	153008 (27.6)	155056 (27.6)	0.000
Current smoker	35111 (23.1)	111998 (26.6)	141002 (25.4)	144500 (25.7)	0.007
Past smoker	72559 (47.8)	195528 (46.4)	260599 (47.0)	263129 (46.8)	0.006
**Alcohol drinker – no. (%)**					
Yes	92697 (61.1)	261221 (62.0)	344124 (62.1)	345638 (61.4)	0.014
No	45266 (29.8)	119770 (28.4)	157051 (28.3)	164181 (29.2)	0.023
Unknown	13844 (9.1)	40223 (9.6)	53433 (9.6)	52867 (9.4)	
**Medication[e] – no. (%)**					
Alpha-blocker	17089 (11.3)	38166 (9.1)	56547 (10.2)	54990 (9.8)	0.001
Oral anticoagulant agent	13055 (8.6)	34986 (8.3)	47457 (8.6)	47408 (8.4)	0.009
Antiplatelet agent	13365 (8.8)	40980 (9.7)	53907 (9.7)	53865 (9.6)	0.007
Aspirin	50393 (33.2)	148837 (35.3)	196084 (35.4)	196731 (35.0)	0.003
Beta-blocker	47734 (31.4)	135829 (32.3)	181804 (32.8)	181431 (32.2)	0.009

Table C Baseline characteristics and standardised differences of trial-eligible patients after applying trial criteria included in inverse-probability—weighted analysis before and after weighting for the reference trial emulation

**Characteristic**	**Before weighting**		**After weighting**		
	**ARB** N=151,807	**ACEi** N=421,214	**ARB** N=554,609	**ACEi** N=562,686	**SMD**
Calcium-channel blocker	52535 (34.6)	135915 (32.3)	189502 (34.2)	186885 (33.2)	0.010
Digoxin	5430 (3.6)	16976 (4.0)	20687 (3.7)	22649 (4.0)	0.015
Diuretics	63949 (42.1)	163355 (38.8)	228374 (41.2)	225328 (40.1)	0.015
Diabetic treatment	38321 (25.2)	101647 (24.1)	138112 (24.9)	138250 (24.6)	0.001
Nitrates	14102 (9.3)	43919 (10.4)	57489 (10.4)	57559 (10.2)	0.007
Statins	79774 (52.6)	225469 (53.5)	296624 (53.5)	299874 (53.3)	0.002
**Time-related variables – mean (SD)**					
Time since trial-eligible period	441.8 (1001.9)	269.9 (760.8)	354.8 (906.2)	312.7 (830.7)	0.006
Number of prior ARB eligible periods	0.9 (1.3)	0.1 (0.5)	0.4 (0.9)	0.3 (0.9)	0.024
Number of prior ACEi eligible periods	1.4 (2.5)	1.5 (2.4)	1.5 (2.5)	1.5 (2.4)	0.029
Calendar year	2011 (5.4)	2010 (5.3)	2010 (5.4)	2010 (5.3)	0.008
**Healthcare utilisation[f] - mean (SD)**					
Number of GP appointments	5.8 (23.2)	9.9 (28.5)	8.5 (28.2)	8.9 (27.1)	0.001
Number of hospital admissions	2.9 (10.7)	3.1 (10.4)	3.3 (11.1)	3.2 (10.8)	0.024
**Index of multiple deprivation – no. (%)**					
1 (least)	30758 (20.3)	81277 (19.3)	107889 (19.5)	109527 (19.5)	0.002
2	32055 (21.1)	86909 (20.6)	114994 (20.7)	116757 (20.8)	0.001
3	30044 (19.8)	82898 (19.7)	109518 (19.8)	110932 (19.7)	0.002
4	30343 (20.0)	85793 (20.4)	112392 (20.3)	114106 (20.3)	0.000
5 (most)	28607 (18.8)	84337 (20.0)	109817 (19.8)	111365 (19.8)	0.002

N= number of patients; no. (%)=number (percent); SD= standard deviation; SMD=standardised mean difference; BP= blood pressure; CAD=coronary artery disease; PAD=peripheral artery disease

Post-weighting N displays weighted distribution of number of patients in the two exposure groups.

Inverse-probability weights are unstabilized.

One third of ONTARGET participants received both ramipril plus telmisartan.

Table C Baseline characteristics and standardised differences of trial-eligible patients after applying trial criteria included in inverse-probability—weighted analysis before and after weighting for the reference trial emulation

Characteristic	Before weighting		After weighting		
	**ARB**<br>N=151,807	**ACEi**<br>N=421,214	**ARB**<br>N=554,609	**ACEi**<br>N=562,686	**SMD**

[a] Includes diagnosis of: MI at least 2 days prior, angina at least 30 days prior, angioplasty at least 30 days prior, CABG at least 4 years prior

[b] Includes diagnosis of: stroke/TIA

[c] Includes diagnosis of: limb bypass surgery, limb/foot amputation, intermittent claudication

[d] Includes DM with: retinopathy, neuropathy, chronic kidney disease, proteinuria or other complication

[e] Within 3 months prior to eligible start date. Antiplatelet agent= clopidogrel/ticlopidine

[f] Within 6 months prior to eligible start date.

Table D Baseline characteristics and standardised differences of trial-eligible Black patients after applying trial criteria included in inverse-probability—weighted analysis before and after weighting for extending analysis to underrepresented groups

Characteristic	Black ethnic group				
	Before weighting		After weighting		
	ARB N=11,646	ACEi N=5,947	ARB N=17,144	ACEi N=17,371	SMD
**Age (year) – mean (SD)**	68.9 (8.9)	68.5 (8.9)	68.7 (8.9)	68.7 (8.9)	0.001
**Systolic BP (mmHg) – mean (SD)**	144.8 (19.3)	144.3 (19.0)	145.1 (19.6)	144.8 (19.1)	0.016
**Diastolic BP (mmHg) – mean (SD)**	80.2 (10.8)	80.7 (10.8)	80.7 (10.8)	80.6 (10.8)	0.003
**Body-mass index – mean (SD)**	30.5 (5.9)	29.8 (5.7)	30.2 (5.8)	30.1 (5.7)	0.016
**Creatinine (µmol/l) – mean (SD)**	99.0 (32.5)	97.0 (29.2)	98.2 (31.1)	98.0 (30.6)	0.005
**Female sex – no. (%)**	3439 (57.8)	5982 (51.4)	9247 (53.9)	9314 (53.6)	0.006
**Clinical history – no. (%)**					
CAD[a]	3883 (65.3)	7812 (67.1)	11318 (66.0)	11511 (66.3)	0.005
Cerebrovascular disease[b]	586 (9.9)	1169 (10.0)	1736 (10.1)	1738 (10.0)	0.004
PAD[c]	551 (9.3)	1029 (8.8)	1539 (9.0)	1550 (8.9)	0.002
Diabetes	4623 (77.7)	9114 (78.3)	13397 (78.2)	13575 (78.1)	0.000
High-risk diabetes[d]	3537 (59.5)	6763 (58.1)	10159 (59.3)	10249 (59.0)	0.005
**Smoking status – no. (%)**					
Non-smoker	2489 (41.9)	4789 (41.1)	7080 (41.3)	7165 (41.2)	0.001
Current smoker	1186 (19.9)	2709 (23.3)	3787 (22.1)	3858 (22.2)	0.003
Past smoker	2272 (38.2)	4148 (35.6)	6277 (36.6)	6349 (36.6)	0.001
**Alcohol drinker – no. (%)**					
Yes	2639 (44.4)	5361 (46.0)	7789 (45.4)	7908 (45.5)	0.002
No	2836 (47.7)	5323 (45.7)	7911 (46.1)	8068 (46.4)	0.006
Unknown	472 (7.9)	962 (8.3)	1444 (8.4)	1395 (8.0)	0.014
**Medication[e] – no. (%)**					
Alpha-blocker	1090 (18.3)	1657 (14.2)	2856 (16.7)	2799 (16.1)	0.015
Oral anticoagulant agent	211 (3.6)	390 (3.4)	604 (3.5)	596 (3.4)	0.005
Antiplatelet agent	334 (5.6)	630 (5.4)	942 (5.5)	974 (5.6)	0.005
Aspirin	1638 (27.5)	3167 (27.2)	4884 (28.5)	4843 (27.9)	0.013
Beta-blocker	1329 (22.4)	2459 (21.1)	3842 (22.4)	3789 (21.8)	0.014
Calcium-channel blocker	3029 (50.9)	5616 (48.2)	8783 (51.2)	8636 (49.7)	0.030
Digoxin	64 (1.1)	114 (0.9)	181 (1.1)	164 (1.0)	0.011
Diuretics	2470 (41.5)	4252 (36.5)	6895 (40.2)	6777 (39.0)	0.025

Table D Baseline characteristics and standardised differences of trial-eligible Black patients after applying trial criteria included in inverse-probability—weighted analysis before and after weighting for extending analysis to underrepresented groups

**Characteristic**	**Black ethnic group**				
	**Before weighting**		**After weighting**		
	**ARB** N=11,646	**ACEi** N=5,947	**ARB** N=17,144	**ACEi** N=17,371	**SMD**
Diabetic treatment	2486 (41.8)	4992 (42.9)	7414 (43.2)	7461 (43.0)	0.006
Nitrates	314 (5.3)	589 (5.1)	869 (5.1)	867 (5.0)	0.004
Statins	2700 (45.4)	5350 (45.9)	8086 (47.2)	8067 (46.4)	0.015
**Time-related variables – mean (SD)**					
Time since trial-eligible period	430.7 (949.1)	280.8 (764.7)	368.9 (891.5)	346.0 (864.9)	0.027
Number of prior ARB eligible periods	1.2 (2.3)	0.3 (1.0)	0.7 (1.9)	0.6 (1.6)	0.023
Number of prior ACEi eligible periods	1.3 (2.4)	1.4 (2.3)	1.4 (2.5)	1.4 (2.4)	0.090
Calendar year	2011 (5.3)	2011 (5.3)	2011 (5.4)	2011 (5.3)	0.002
**Healthcare utilisation[f] – mean (SD)**					
Number of GP appointments	7.5 (27.5)	12.4 (34.6)	10.4 (32.0)	10.7 (32.5)	0.010
Number of hospital admissions	3.0 (12.6)	2.8 (11.2)	3.0 (12.4)	3.1 (11.9)	0.010
**Index of multiple deprivation – no. (%)**					
1 (least)	150 (2.5)	314 (2.7)	433 (2.5)	444 (2.6)	0.002
2	370 (6.2)	675 (5.8)	989 (5.8)	1011 (5.8)	0.002
3	924 (15.5)	1735 (14.9)	2609 (15.2)	2649 (15.3)	0.001
4	2131 (35.8)	4250 (36.5)	6228 (36.3)	6317 (36.4)	0.001
5 (most)	2372 (39.9)	4672 (40.1)	6885 (40.2)	6950 (40.0)	0.003

N= number of patients; no. (%)=number (percent); SD= standard deviation; SMD=standardised mean difference; BP= blood pressure; CAD=coronary artery disease; PAD=peripheral artery disease

Post-weighting N displays weighted distribution of number of patients in the two exposure groups.

Inverse-probability weights are unstabilized.

One third of ONTARGET participants received both ramipril plus telmisartan.

[a] Includes diagnosis of: MI at least 2 days prior, angina at least 30 days prior, angioplasty at least 30 days prior, CABG at least 4 years prior

[b] Includes diagnosis of: stroke/TIA

Table D Baseline characteristics and standardised differences of trial-eligible Black patients after applying trial criteria included in inverse-probability—weighted analysis before and after weighting for extending analysis to underrepresented groups

**Characteristic**	**Black ethnic group**				
	**Before weighting**		**After weighting**		
	**ARB** N=11,646	**ACEi** N=5,947	**ARB** N=17,144	**ACEi** N=17,371	**SMD**

[c] Includes diagnosis of: limb bypass surgery, limb/foot amputation, intermittent claudication

[d] Includes DM with: retinopathy, neuropathy, chronic kidney disease, proteinuria or other complication

[e] Within 3 months prior to eligible start date. Antiplatelet agent= clopidogrel/ticlopidine

[f] Within 6 months prior to eligible start date.

Table E Baseline characteristics and standardised differences of trial-eligible South Asian patients after applying trial criteria included in inverse-probability—weighted analysis before and after weighting for extending analysis to underrepresented groups

**Characteristic**	**South Asian ethnic group**				
	**Before weighting**		**After weighting**		
	**ARB** N=10,668	**ACEi** N=20,137	**ARB** N=30,176	**ACEi** N=30,309	**SMD**
**Age (year) – mean (SD)**	67.7 (845)	67.1 (8.5)	67.4 (8.5)	67.4 (8.5)	0.006
**Systolic BP (mmHg) – mean (SD)**	140.4 (19.1)	141.0 (19.3)	141.2 (19.4)	141.1 (19.3)	0.008
**Diastolic BP (mmHg) – mean (SD)**	78.0 (10.7)	78.7 (10.8)	78.6 (10.8)	78.6 (10.8)	0.004
**Body-mass index – mean (SD)**	28.1 (5.0)	27.6 (5.0)	27.9 (4.9)	27.8 (5.1)	0.018
**Creatinine (µmol/l) – mean (SD)**	91.3 (31.2)	88.0 (28.2)	90.2 (29.4)	90.2 (30.0)	0.001
**Female sex – no. (%)**	5561 (52.1)	9362 (46.5)	14662 (48.6)	14678 (48.4)	0.003
**Clinical history – no. (%)**					
CAD[a]	7132 (66.9)	13740 (68.2)	20362 (67.5)	20557 (67.8)	0.007
Cerebrovascular disease[b]	1005 (9.4)	1986 (9.9)	2873 (9.5)	2907 (9.6)	0.002
PAD[c]	982 (9.2)	1828 (9.1)	2751 (9.1)	2755 (9.1)	0.001
Diabetes	8446 (79.2)	15767 (78.3)	23771 (78.8)	23812 (78.6)	0.005
High-risk diabetes[d]	6068 (56.9)	10877 (54.0)	16878 (55.9)	16741 (55.2)	0.014
**Smoking status – no. (%)**					
Non-smoker	4828 (45.3)	8806 (43.7)	13312 (44.1)	13318 (43.9)	0.004
Current smoker	2193 (20.6)	4648 (23.1)	6753 (22.4)	6821 (22.5)	0.003
Past smoker	3647 (34.2)	6683 (33.2)	10111 (33.5)	10170 (33.6)	0.001
**Alcohol drinker – no. (%)**					
Yes	3251 (30.5)	6273 (31.2)	9357 (31.0)	9375 (30.9)	0.002
No	6488 (60.8)	12033 (59.8)	17776 (58.9)	18259 (60.2)	0.027
Unknown	929 (8.7)	1831 (9.1)	2823 (9.4)	2675 (8.8)	0.018
**Medication[e] – no. (%)**					
Alpha-blocker	1263 (11.8)	1860 (9.2)	3203 (10.6)	3117 (10.3)	0.011
Oral anticoagulant agent	337 (3.2)	553 (2.8)	912 (3.0)	875 (2.9)	0.008
Antiplatelet agent	1062 (10.0)	1940 (9.6)	3049 (10.1)	3017 (10.0)	0.005
Aspirin	3902 (36.6)	7546 (37.5)	11425 (37.9)	11461 (37.8)	0.001
Beta-blocker	2986 (28.0)	5486 (27.2)	8563 (28.4)	8436 (27.8)	0.012
Calcium-channel blocker	3757 (35.2)	6430 (31.9)	10408 (34.5)	10195 (33.6)	0.018
Digoxin	115 (1.1)	230 (1.1)	344 (1.1)	351 (1.2)	0.002
Diuretics	3395 (31.8)	5319 (26.4)	9061 (30.0)	8744 (28.9)	0.026

Table E Baseline characteristics and standardised differences of trial-eligible South Asian patients after applying trial criteria included in inverse-probability—weighted analysis before and after weighting for extending analysis to underrepresented groups

**Characteristic**	**South Asian ethnic group**				
	**Before weighting**		**After weighting**		
	**ARB** N=10,668	**ACEi** N=20,137	**ARB** N=30,176	**ACEi** N=30,309	**SMD**
Diabetic treatment	5188 (48.6)	9749 (48.4)	14766 (48.9)	14750 (48.7)	0.005
Nitrates	1131 (10.6)	2154 (10.7)	3302 (10.9)	3281 (10.8)	0.004
Statins	6489 (60.8)	12055 (59.9)	18415 (61.0)	18355 (60.6)	0.010
**Time-related variables – mean (SD)**					
Time since trial-eligible period	429.3 (954.2)	274.8 (760.9)	355.9 (884.3)	337.6 (856.5)	0.021
Number of prior ARB eligible periods	1.0 (1.6)	0.2 (0.8)	0.6 (1.2)	0.5 (1.3)	0.088
Number of prior ACEi eligible periods	1.2 (1.9)	1.2 (2.0)	1.3 (2.0)	1.3 (2.1)	0.001
Calendar year	2012 (5.2)	2011 (5.2)	2011 (5.3)	2011 (5.1)	0.001
**Healthcare utilisation[f] – mean (SD)**					
Number of GP appointments	7.1 (27.4)	10.7 (32.7)	9.6 (31.9)	9.5 (31.2)	0.003
Number of hospital admissions	3.1 (12.0)	3.0 (11.8)	3.2 (12.3)	3.2 (12.3)	0.004
**Index of multiple deprivation – no. (%)**					
1 (least)	1149 (10.8)	2004 (10.0)	3047 (10.1)	3088 (10.2)	0.003
2	1603 (15.0)	2708 (13.5)	4223 (14.0)	4239 (14.0)	0.000
3	2323 (21.8)	4166 (20.7)	6350 (21.0)	6440 (21.3)	0.005
4	2957 (27.7)	5802 (28.8)	8516 (28.2)	8575 (28.3)	0.002
5 (most)	2636 (24.7)	5457 (27.1)	8040 (26.6)	7966 (26.3)	0.008

N= number of patients; no. (%)=number (percent); SD= standard deviation; SMD=standardised mean difference; BP= blood pressure; CAD=coronary artery disease; PAD=peripheral artery disease

Post-weighting N displays weighted distribution of number of patients in the two exposure groups.

Inverse-probability weights are unstabilized.

One third of ONTARGET participants received both ramipril plus telmisartan.

[a] Includes diagnosis of: MI at least 2 days prior, angina at least 30 days prior, angioplasty at least 30 days prior, CABG at least 4 years prior

[b] Includes diagnosis of: stroke/TIA

Table E Baseline characteristics and standardised differences of trial-eligible South Asian patients after applying trial criteria included in inverse-probability—weighted analysis before and after weighting for extending analysis to underrepresented groups

**Characteristic**	**South Asian ethnic group**				
	**Before weighting**		**After weighting**		
	**ARB** N=10,668	**ACEi** N=20,137	**ARB** N=30,176	**ACEi** N=30,309	**SMD**

[c] Includes diagnosis of: limb bypass surgery, limb/foot amputation, intermittent claudication

[d] Includes DM with: retinopathy, neuropathy, chronic kidney disease, proteinuria or other complication

[e] Within 3 months prior to eligible start date. Antiplatelet agent= clopidogrel/ticlopidine

[f] Within 6 months prior to eligible start date.

Table F Baseline characteristics and standardised differences of trial-eligible White patients after applying trial criteria included in inverse-probability—weighted analysis before and after weighting for extending analysis to underrepresented groups

**Characteristic**	**White ethnic group**				
	**Before weighting**		**After weighting**		
	**ARB** N=135,192	**ACEi** N=389,431	**ARB** N=507,289	**ACEi** N=515,006	**SMD**
**Age (year) – mean (SD)**	71.7 (9.1)	71.1 (9.4)	71.4 (9.3)	71.3 (9.3)	0.008
**Systolic BP (mmHg) – mean (SD)**	144.2 (20.3)	143.8 (20.3)	144.9 (20.6)	144.2 (20.4)	0.033
**Diastolic BP (mmHg) – mean (SD)**	78.8 (10.9)	79.1 (11.0)	79.3 (11.1)	79.1 (11.0)	0.016
**Body-mass index – mean (SD)**	29.2 (5.8)	28.8 (5.8)	29.0 (5.8)	28.9 (5.9)	0.010
**Creatinine (µmol/l) – mean (SD)**	94.1 (29.2)	93.0 (207.2)	93.9 (28.4)	93.5 (27.9)	0.013
**Female sex – no. (%)**	73849 (54.6)	187167 (48.1)	255003 (50.3)	256177 (49.7)	0.011
**Clinical history – no. (%)**					
CAD[a]	94954 (70.2)	277145 (71.2)	357856 (70.5)	364655 (70.8)	0.006
Cerebrovascular disease[b]	14480 (10.7)	41666 (10.7)	53996 (10.6)	54866 (10.7)	0.000
PAD[c]	12625 (9.3)	36101 (9.3)	46869 (9.2)	47714 (9.3)	0.001
Diabetes	80640 (59.7)	225149 (57.8)	296114 (58.4)	299909 (58.2)	0.003
High-risk diabetes[d]	66692 (49.3)	179831 (46.2)	242995 (47.9)	243302 (47.2)	0.013
**Smoking status – no. (%)**					
Non-smoker	36820 (27.2)	100093 (25.7)	132616 (26.1)	134574 (26.1)	0.000
Current smoker	31732 (23.5)	104641 (26.9)	130463 (25.7)	133821 (26.0)	0.009
Past smoker	66640 (49.3)	184697 (47.4)	244211 (48.1)	246610 (47.9)	0.005
**Alcohol drinker – no. (%)**					
Yes	86807 (64.2)	249587 (64.1)	326979 (64.5)	328355 (63.8)	0.015
No	35942 (26.6)	102414 (26.3)	131144 (25.9)	137854 (26.8)	0.021
Unknown	12443 (9.2)	37430 (9.6)	49167 (9.7)	48797 (9.5)	0.007

Table F Baseline characteristics and standardised differences of trial-eligible White patients after applying trial criteria included in inverse-probability—weighted analysis before and after weighting for extending analysis to underrepresented groups

Characteristic	White ethnic group				
	Before weighting		After weighting		
	ARB N=135,192	ACEi N=389,431	ARB N=507,289	ACEi N=515,006	SMD
**Medication[e] – no. (%)**					
Alpha-blocker	14736 (10.9)	34649 (8.9)	50488 (10.0)	49073 (9.5)	0.014
Oral anticoagulant agent	12507 (9.3)	34043 (8.7)	45941 (9.1)	45937 (8.9)	0.005
Antiplatelet agent	11969 (8.9)	38410 (9.9)	49916 (9.8)	49874 (9.7)	0.005
Aspirin	44853 (33.2)	138124 (35.5)	179775 (35.4)	180427 (35.0)	0.008
Beta-blocker	43419 (32.1)	127884 (32.8)	169400 (33.4)	169205 (32.9)	0.011
Calcium-channel blocker	45749 (33.8)	123869 (31.8)	170311 (33.6)	168053 (32.6)	0.020
Digoxin	5251 (3.9)	16636 (4.3)	20162 (4.0)	22133 (4.3)	0.016
Diuretics	58084 (43.0)	153784 (39.5)	212419 (41.9)	209807 (40.7)	0.023
Diabetic treatment	30647 (22.7)	86906 (22.3)	115932 (22.9)	116039 (22.5)	0.008
Nitrates	12657 (9.4)	41176 (10.6)	53318 (10.5)	53411 (10.4)	0.005
Statins	70585 (52.2)	208064 (53.4)	270123 (53.3)	272453 (53.1)	0.003
**Time-related variables – mean (SD)**					
Time since trial-eligible period	443.3 (1007.9)	269.3 (760.7)	354.3 (907.9)	310.1 (827.9)	0.049
Number of prior ARB eligible periods	0.8 (1.2)	0.1 (0.5)	0.3 (0.8)	0.3 (0.9)	0.000
Number of prior ACEi eligible periods	1.4 (2.5)	1.5 (2.4)	1.5 (2.5)	1.5 (2.5)	0.073
Calendar year	2010 (5.4)	2010 (5.3)	2010 (5.4)	2010 (5.3)	0.007
**Healthcare utilisation[f] – mean (SD)**					
Number of GP appointments	5.6 (22.6)	9.7 (28.0)	8.3 (27.8)	8.8 (26.7)	0.020
Number of hospital admissions	2.9 (10.5)	3.1 (10.3)	3.3 (11.0)	3.2 (10.7)	0.014

Table F Baseline characteristics and standardised differences of trial-eligible White patients after applying trial criteria included in inverse-probability—weighted analysis before and after weighting for extending analysis to underrepresented groups

**Characteristic**	**White ethnic group**				
	**Before weighting**		**After weighting**		
	**ARB** N=135,192	**ACEi** N=389,431	**ARB** N=507,289	**ACEi** N=515,006	**SMD**
**Index of multiple deprivation – no. (%)**					
1 (least)	29459 (21.8)	78959 (20.3)	104409 (20.6)	105995 (20.6)	0.000
2	30082 (22.3)	83526 (21.5)	109782 (21.6)	111506 (21.7)	0.000
3	26797 (19.8)	76997 (19.8)	100559 (19.8)	101842 (19.8)	0.001
4	25255 (18.7)	75741 (19.5)	97647 (19.3)	99214 (19.3)	0.000
5 (most)	23599 (17.5)	74208 (19.1)	94892 (18.7)	96448 (18.7)	0.001

N= number of patients; no. (%)=number (percent); SD= standard deviation; SMD=standardised mean difference; BP= blood pressure; CAD=coronary artery disease; PAD=peripheral artery disease

Post-weighting N displays weighted distribution of number of patients in the two exposure groups.

Inverse-probability weights are unstabilized.

One third of ONTARGET participants received both ramipril plus telmisartan.

[a] Includes diagnosis of: MI at least 2 days prior, angina at least 30 days prior, angioplasty at least 30 days prior, CABG at least 4 years prior

[b] Includes diagnosis of: stroke/TIA

[c] Includes diagnosis of: limb bypass surgery, limb/foot amputation, intermittent claudication

[d] Includes DM with: retinopathy, neuropathy, chronic kidney disease, proteinuria or other complication

[e] Within 3 months prior to eligible start date. Antiplatelet agent= clopidogrel/ticlopidine

[f] Within 6 months prior to eligible start date.

Table G Number of events for the primary outcome, its components, main secondary outcome and death from any cause for ARB vs ACEi using an inverse-probability—weighted analysis of trial-eligible patients in CPRD Aurum after emulation and benchmarking findings against the reference trial (ONTARGET)

**Outcome**	**CPRD**			**ONTARGET**
	**ARB** (N=151,807)	**ACEi** (N=421,214)	**ARB vs ACEi** (N=573,021)	**Telmisartan vs ramipril** (N=17,118)
	Number (percent)		*Hazard ratio (95% CI)*	
Primary composite	27327 (18.0)	80624 (19.1)	0.96 (0.95, 0.98)	1.01 (0.94, 1.09)
Main secondary outcome	21673 (14.3)	65908 (15.7)	0.94 (0.92, 0.96)	0.99 (0.91, 1.07)
Myocardial infarction	9913 (6.5)	31069 (7.4)	0.96 (0.94, 0.99)	1.07 (0.94, 1.22)
Stroke	6870 (4.5)	19390 (4.6)	0.96 (0.93, 0.99)	0.91 (0.79, 1.05)
Hospitalisation for heart failure	10029 (6.6)	26771 (6.4)	1.03 (1.00, 1.06)	1.12 (0.97, 1.29)
Death from cardiovascular causes	9199 (6.1)	28355 (6.7)	0.91 (0.89, 0.94)	1.00 (0.89, 1.12)
Death from non-cardiovascular causes	14241 (9.4)	43391 (10.3)	0.91 (0.89, 0.93)	0.96 (0.83, 1.10)
Death from any cause	23440 (15.4)	71743 (17.0)	0.91 (0.90, 0.93)	0.98 (0.90, 1.07)
Doubling of serum creatinine	5289 (3.9)	12588 (3.4)	1.09 (1.05, 1.13)	1.11 (0.88, 1.39)

Primary composite outcome: death from cardiovascular causes, myocardial infarction, stroke, or hospitalisation for heart failure.

Main secondary outcome: death from cardiovascular causes, myocardial infarction, or stroke.

ESKD: end-stage kidney disease; GFR: glomerular filtration rate.

CPRD weighted analysis includes 1 randomly selected trial-eligible period per patient. Inverse-probability—weighted with robust standard errors.

Myocardial infarction and stroke include both fatal and non-fatal events.

Only outcomes studied in ONTARGET were studied for the benchmarking analysis.

ONTARGET results are from published findings.

Table H Comparative effectiveness of ARB vs ACEi for the primary and secondary outcomes overall and by ethnicity with a test for multiplicative heterogeneity using an inverse-probability—weighted analysis of trial-eligible patients in CPRD Aurum with an on-treatment approach.

**Outcome**	**Overall** (N=573,021)	**By ethnic group**			
		**Black** (N=17,593)	**South Asian** (N=30,805)	**White** (N=524,624)	**P value for interaction**
	Hazard ratio (95% CI)				
Primary composite	0.95 (0.94, 0.97)	1.00 (0.91, 1.10)	0.95 (0.89, 1.02)	0.95 (0.94, 0.97)	0.581
Main secondary outcome	0.93 (0.92, 0.95)	1.01 (0.90, 1.12)	0.96 (0.90, 1.03)	0.93 (0.91, 0.95)	0.236
Myocardial infarction	0.95 (0.92, 0.98)	1.10 (0.92, 1.32)	0.96 (0.88, 1.06)	0.95 (0.92, 0.97)	0.245
Stroke	0.95 (0.92, 0.98)	0.95 (0.81, 1.11)	0.97 (0.85, 1.10)	0.95 (0.92, 0.98)	0.975
Hospitalisation for heart failure	1.01 (0.99, 1.04)	0.98 (0.85, 1.13)	0.97 (0.87, 1.07)	1.02 (0.99, 1.05)	0.579
Death from cardiovascular causes	0.90 (0.88, 0.93)	1.14 (0.97, 1.35)	0.94 (0.83, 1.06)	0.89 (0.87, 0.92)	0.013
Death from non-cardiovascular causes	0.90 (0.88, 0.92)	0.97 (0.85, 1.12)	0.91 (0.82, 1.02)	0.90 (0.88, 0.92)	0.571
Death from any cause	0.90 (0.89, 0.92)	1.04 (0.93, 1.15)	0.92 (0.85, 1.00)	0.90 (0.88, 0.92)	0.026
Loss of GFR or ESKD	1.05 (1.02, 1.08)	1.09 (0.94, 1.26)	1.00 (0.90, 1.13)	1.05 (1.02, 1.08)	0.665
ESKD	0.99 (0.94, 1.05)	1.10 (0.88, 1.37)	0.96 (0.80, 1.14)	0.99 (0.94, 1.05)	0.603
Doubling of serum creatinine	1.06 (1.02, 1.10)	1.08 (0.89, 1.29)	1.02 (0.88, 1.76)	1.06 (1.02, 1.10)	0.852
Angioedema	0.62 (0.50, 0.77)	0.36 (0.18, 0.71)	0.60 (0.25, 1.44)	0.66 (0.52, 0.84)	0.254

Primary composite outcome: death from cardiovascular causes, myocardial infarction, stroke, or hospitalisation for heart failure. Main secondary outcome: death from cardiovascular causes, myocardial infarction, or stroke. Loss of GFR or ESKD defined as: 50% reduction in estimated glomerular filtration ratio (eGFR), start of kidney replacement therapy (KRT) or eGFR<15ml/min/1.73m^2. ESKD defined as: start of KRT or eGFR<15ml/min/1.73m^2.

ESKD: end-stage kidney disease; GFR: glomerular filtration rate.

CPRD weighted analysis includes 1 randomly selected trial-eligible period per patient. Inverse-probability—weighted with robust standard errors.

Myocardial infarction and stroke include both fatal and non-fatal events.

Under on-treatment analysis, patients were additionally censored at the end of an eligible period, if they switched treatment or started dual therapy. This was denoted as date of last drug and patients were censored at this date +60 days.

Table I Comparative effectiveness of ARB vs ACEi for the primary and secondary outcomes overall and by ethnicity using an inverse-probability—weighted analysis of trial-eligible patients in CPRD Aurum with after multiple imputation of missing values.

**Outcome**	**Overall** (N=690,166)	**By ethnic group**		
		**Black** (N=21,015)	**South Asian** (N=37,240)	**White** (N=631,911)
	Hazard ratio (95% CI)			
Primary composite	0.95 (0.94, 0.97)	1.02 (0.94, 1.11)	0.99 (0.93, 1.04)	0.95 (0.93, 0.96)
Main secondary outcome	0.93 (0.91, 0.94)	1.01 (0.92, 1.12)	1.00 (0.94, 1.06)	0.92 (0.91, 0.94)
Myocardial infarction	0.95 (0.92, 0.97)	1.08 (0.92, 1.27)	1.00 (0.91, 1.09)	0.94 (0.92, 0.97)
Stroke	0.95 (0.93, 0.98)	1.02 (0.88, 1.17)	0.98 (0.87, 1.10)	0.95 (0.92, 0.98)
Hospitalisation for heart failure	1.03 (1.01, 1.06)	1.01 (0.89, 1.15)	1.00 (0.91, 1.10)	1.03 (1.01, 1.06)
Death from cardiovascular causes	0.90 (0.88, 0.93)	1.13 (0.97, 1.31)	0.99 (0.89, 1.11)	0.89 (0.87, 0.92)
Death from non-cardiovascular causes	0.91 (0.89, 0.93)	0.93 (0.82, 1.06)	0.94 (0.85, 1.03)	0.90 (0.89, 0.92)
Death from any cause	0.91 (0.89, 0.92)	1.01 (0.92, 1.11)	0.96 (0.90, 1.03)	0.90 (0.89, 0.92)
Angioedema	0.62 (0.50, 0.75)	0.37 (0.19, 0.72)	0.73 (0.34, 1.57)	0.65 (0.52, 0.81)

Primary composite outcome: death from cardiovascular causes, myocardial infarction, stroke, or hospitalisation for heart failure. Main secondary outcome: death from cardiovascular causes, myocardial infarction, or stroke. Loss of GFR or ESKD defined as: 50% reduction in estimated glomerular filtration ratio (eGFR), start of kidney replacement therapy (KRT) or eGFR<15ml/min/1.73m^2. ESKD defined as: start of KRT or eGFR<15ml/min/1.73m^2.

ESKD: end-stage kidney disease; GFR: glomerular filtration rate.

CPRD weighted analysis includes 1 randomly selected trial-eligible period per patient. Inverse-probability—weighted with robust standard errors.

Myocardial infarction and stroke include both fatal and non-fatal events.

Multiple imputation of chained equations for missing systolic and diastolic blood pressure and serum creatinine at baseline.

Table J. Table of trial diagnoses (inclusion criteria) and interpretation in CPRD.

**ONTARGET/TRANSCEND**	**CPRD Aurum (HES + ONS Linked)**<br>**READ or ICD 10 code for:**
Aged ≥55 years	Aged ≥55 years prior to prescription of drug
Coronary artery disease	
Previous myocardial infarction (>2 days post uncomplicated MI)	MI at least 2 days prior to prescription of drug
Stable angina or unstable angina >30 days before informed consent and with documented evidence of multivessel coronary artery disease	Angina/stable angina/unstable angina at least 30 days before prescription of drug and previous coronary artery disease diagnosis
Multi-vessel PTCA >30 days before informed consent	Read, ICD-10 or OPCS code for coronary angioplasty at least 30 days before prescription of drug
Multi-vessel CABG surgery >4 years before informed consent, or with recurrent angina following surgery	Read, ICD-10 or OPCS code for CABG at least 4 years before prescription of drug or with angina after CABG
Peripheral artery disease	
Previous limb bypass surgery or angioplasty	Read, ICD-10 or OPCS code for limb bypass surgery or angioplasty
Previous limb or foot amputation	Read, ICD-10 or OPCS code for limb/foot amputation
Intermittent claudication, with ankle:arm BP ratio <=0.80 on at least 1 side	Intermittent claudication
Significant peripheral artery stenosis (>50%) documented by angiography or non-invasive test	Not applicable
Cerebrovascular disease	
Previous stroke	Stroke before prescription of drug
Transient ischemic attacks >7 days and <1 year before informed consent	Transient ischemic attacks before prescription of drug
High-risk diabetes with evidence of end-organ damage	
High-risk diabetes	Specific codes for diabetes with retinopathy, neuropathy, chronic kidney disease or proteinuria

	before prescription of drug or diabetes defined by diabetes codes or diabetes therapy with CKD defined as eGFR<60 or proteinuria defined as ACR>3

Notes: Where dates are used as criteria dates from both CPRD and HES will be used, but if available HES will be preferred.

Table K. Table of trial exclusion criteria and interpretation in CPRD.

**ONTARGET/TRANSCEND exclusion criteria**	**CPRD Aurum (HES + ONS Linked)**<br>**READ or ICD 10 code (prior to eligible for inclusion date, unless otherwise specified) for:**
Inability to discontinue ACEi or ARB	Not applicable
Known hypersensitivity or intolerance to ACEi or ARB	Not applicable
Symptomatic congestive heart failure	Heart failure or left ventricular dysfunction
Hemodynamically significant primary valvular or outflow tract obstruction	Aortic or pulmonary stenosis or previous valve replacement
Constrictive pericarditis	Constrictive pericarditis
Complex congenital heart disease	Congenital heart disease
Syncopal episodes of unknown etiology <3 months before informed consent	Not applicable
Planned cardiac surgery or PTCA <3 months of informed consent	Not applicable
Uncontrolled hypertension on treatment (e.g. BP >160/100 mm Hg)	Last recorded BP >160/100 mmHg for patients on treatment with other antihypertensives prior to ACEi/ARB initiation
Heart transplant recipient	Read, ICD-10 or OPCS code for heart transplant recipient
Stroke due to subarachnoid haemorrhage	Previous cerebral haemorrhage
Significant renal artery disease	Codes for renal artery stenosis or renal artery atherosclerosis; or serum creatinine concentration above 265μmol/L
Hepatic dysfunction	Cirrhosis or other documented liver disease
Uncorrected volume or sodium depletion	Not applicable
Primary hyperaldosteronism	Primary hyperaldosteronism/ Conn's syndrome

Hereditary fructose intolerance	Hereditary fructose intolerance
Other major noncardiac illness expected to reduce life expectancy or interfere with study participation	Recorded solid organ or metastatic malignancy within the last 5 years, drug, alcohol dependence or mental illness.
Simultaneously taking another experimental drug	Not applicable
Significant disability precluding regular follow-up visits	Not applicable
Unable or unwilling to provide written informed consent	Not applicable
Elevated potassium above 5.5mmol/L	Elevated potassium above 5.5mmol/L
Hypotension	SBP <90 mm Hg

Notes: Where dates are used as criteria dates from both CPRD and HES will be used, but if available HES will be preferred. Not applicable used when anticipated there will be extensive missing data or risk of misclassification.

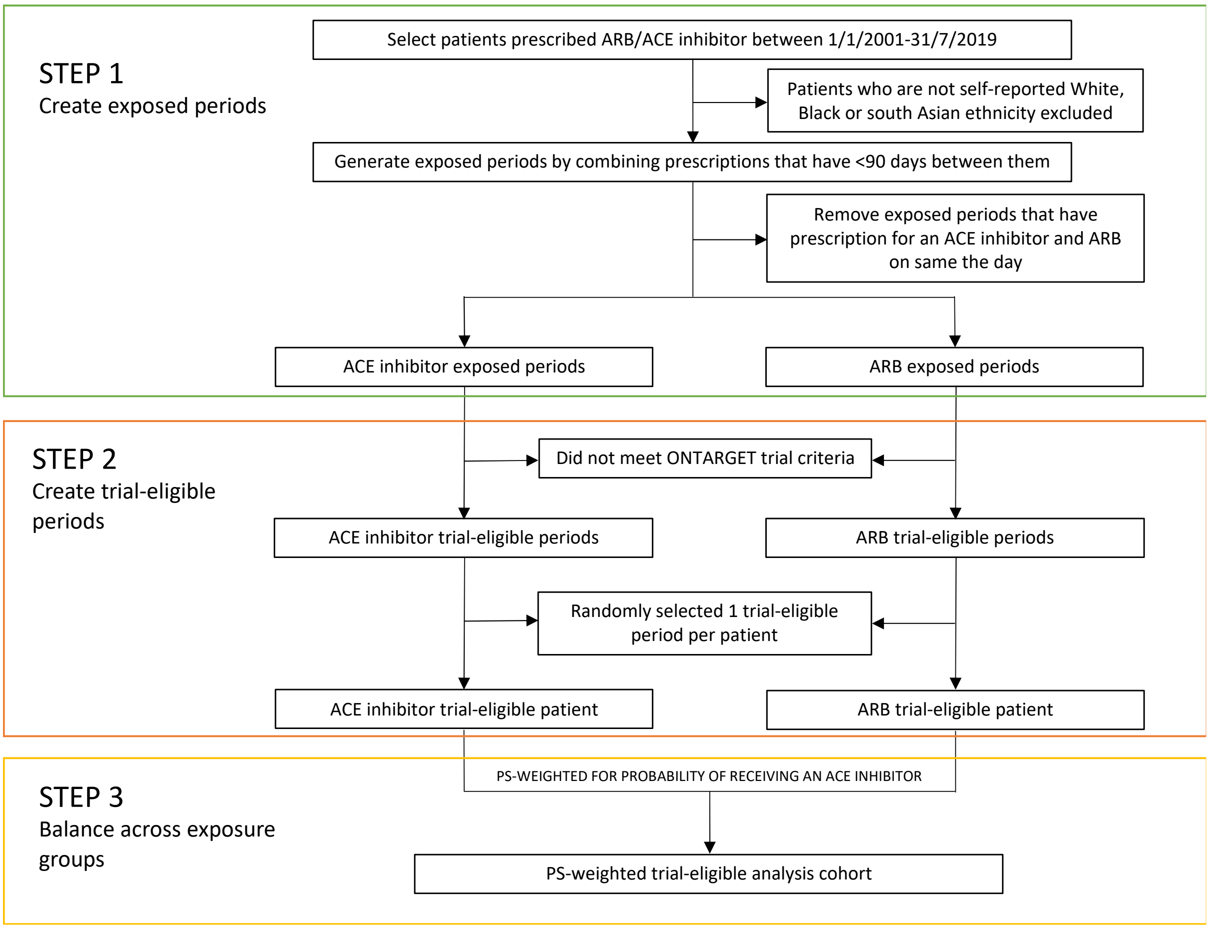

Figure A. Steps to define analysis cohort.

PS=propensity score

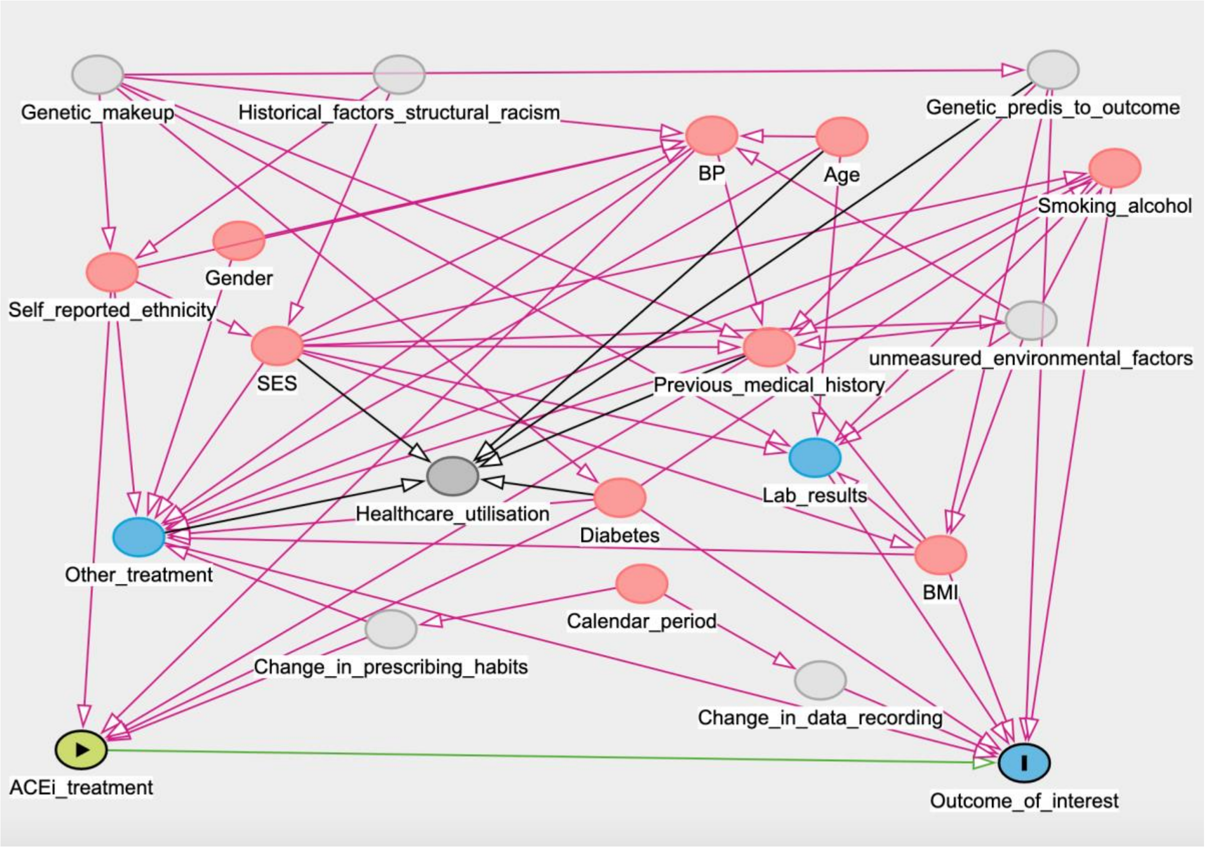

Figure B. Directed acyclic graph to identify confounders for propensity score model
SES: socio-economic status; BP: blood pressure; BMI: body-mass-index.

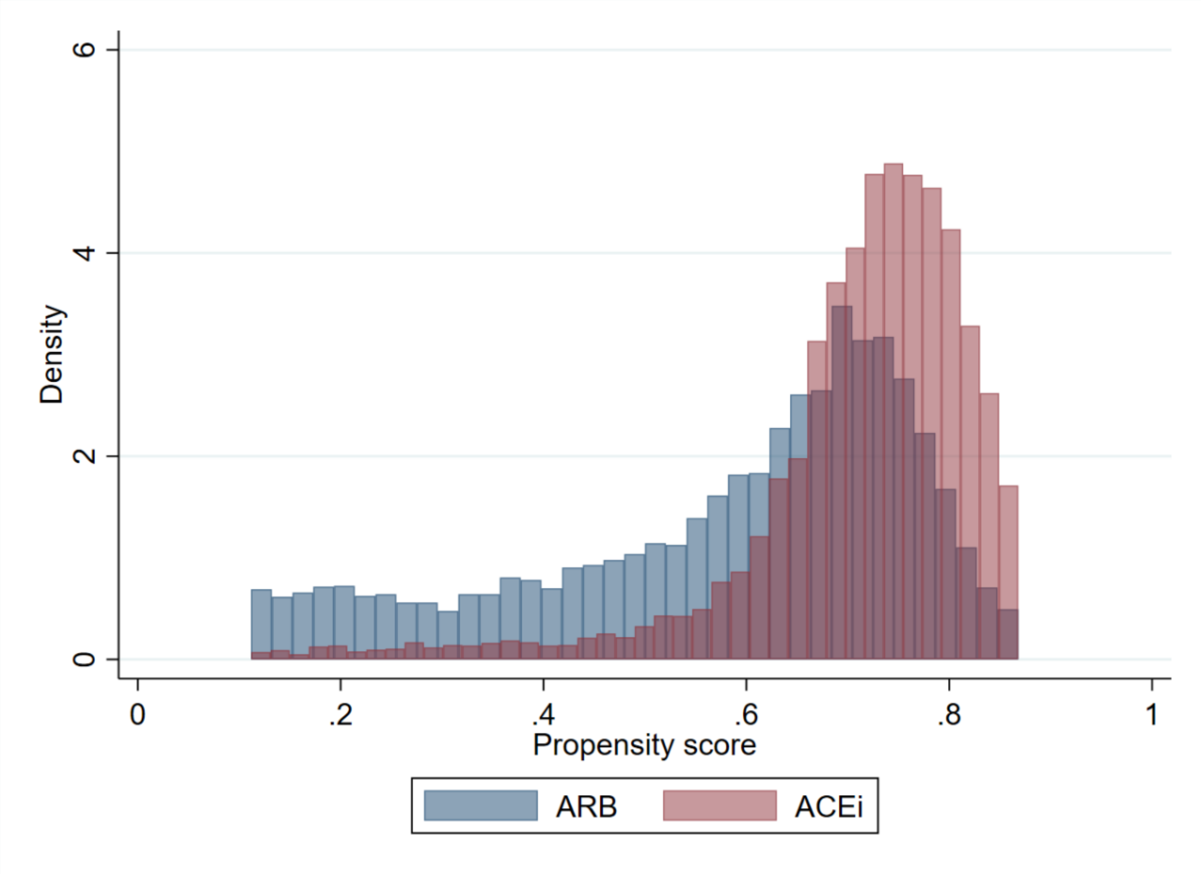

Figure C. Histogram of propensity score distribution after trimming extreme weights for patients of Black ethnicity

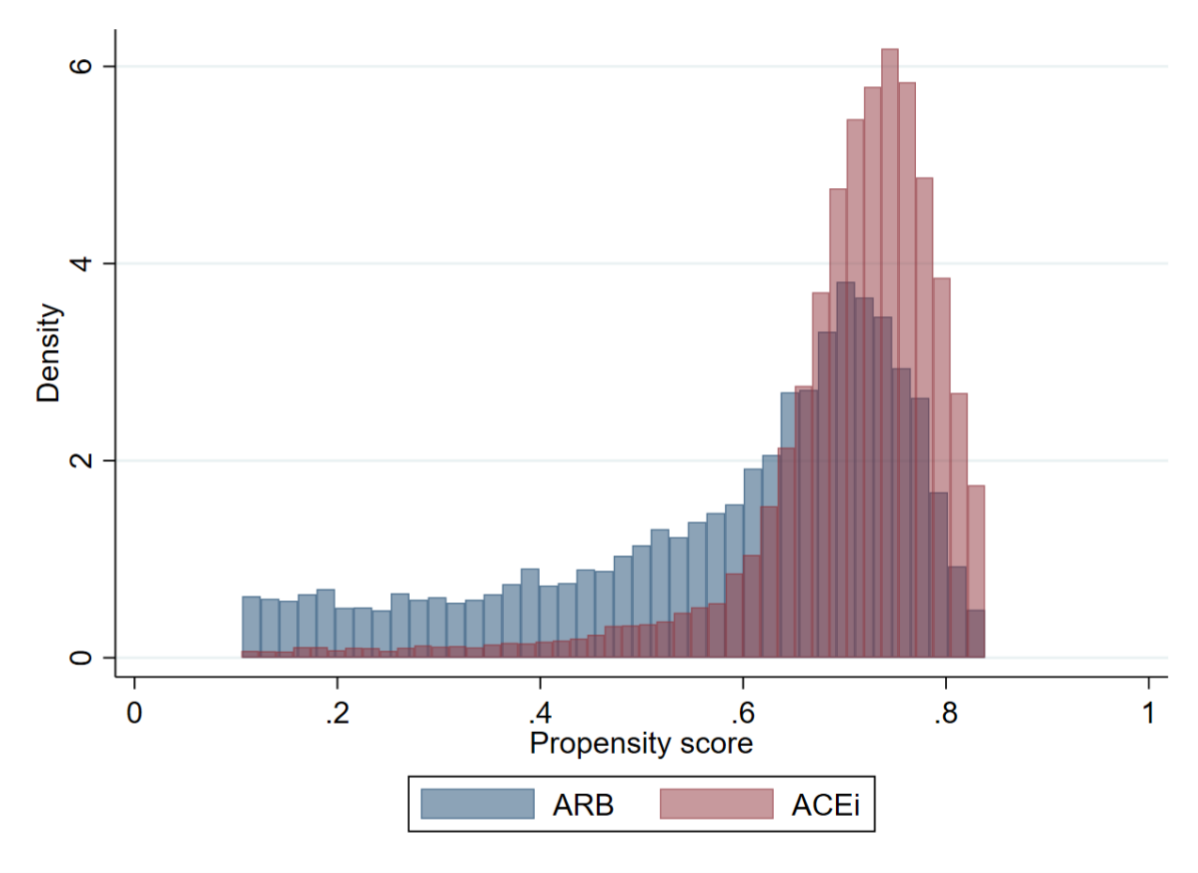

Figure D. Histogram of propensity score distribution after trimming extreme weights for patients of South Asian ethnicity

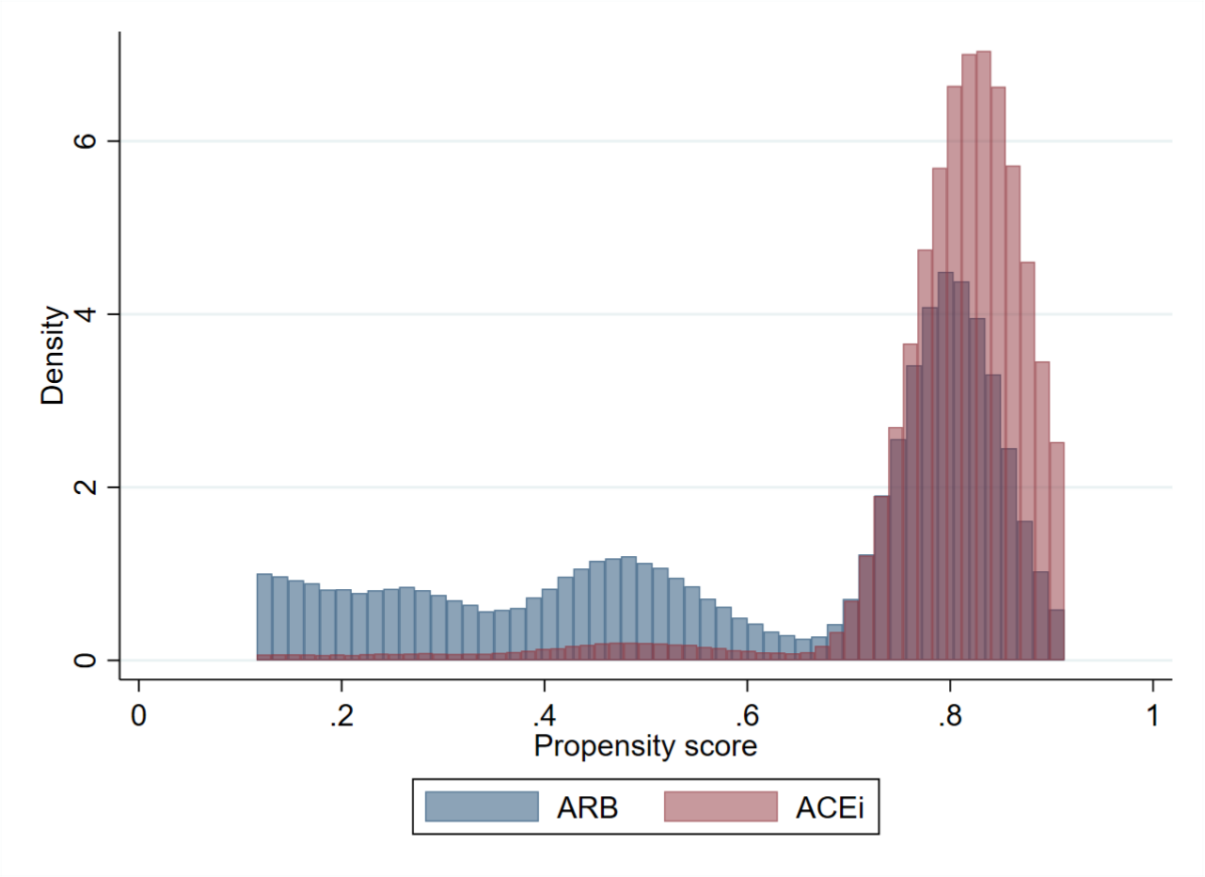

Figure E. Histogram of propensity score distribution after trimming extreme weights for patients of White ethnicity

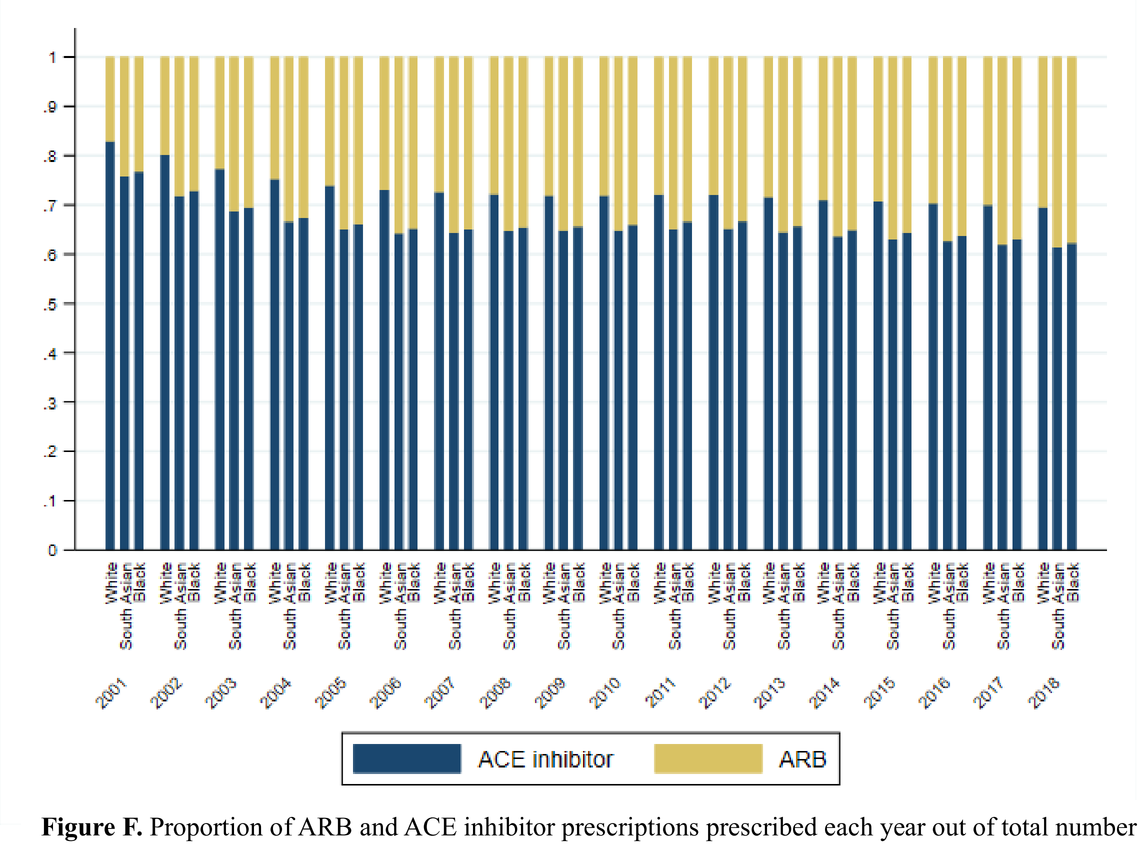

Figure F. Proportion of ARB and ACE inhibitor prescriptions prescribed each year out of total number prescribed within each ethnic group.

Primary outcome (sensitivity	ARB<br>n (%)	ACEi<br>n (%)	ARB vs ACEi<br>HR (95% CI)	P Value for Interaction
On-treatment approach				0.581
Black	843 (14.2)	1607 (13.8)	1.00 (0.91, 1.10)	
South Asian	1682 (15.8)	3323 (16.5)	0.95 (0.89, 1.02)	
White	23675 (17.5)	74707 (19.2)	0.95 (0.94, 0.97)	
Eligible period <2011				0.232
Black	425 (16.4)	850 (14.9)	1.04 (0.92, 1.19)	
South Asian	803 (18.3)	1649 (18.0)	0.98 (0.90, 1.08)	
White	13325 (19.1)	43996 (20.5)	0.94 (0.92, 0.96)	

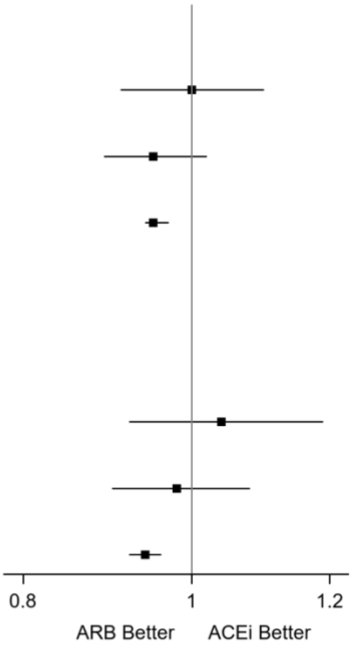

Figure G. Forest plot of sensitivity analysis for extending analysis to underrepresented groups using an inverse-probability—weighted analysis for ARB vs ACEi use for primary composite outcome.
Primary composite outcome is cardiovascular related death, myocardial infarction, stroke, or hospitalisation for heart failure. On-treatment approach censored at treatment discontinuation (i.e., treatment gap of >90 days), switch treatment or start of dual use +60 days. Multiple imputation of missing baseline blood pressure and creatinine using chained equations. Eligible period <2011 is analysis restricted to start of trial-eligible periods prior to 2011. P value is test for heterogeneity using an term interaction between treatment and ethnicity in the Cox proportional hazards model.
