# Supplementary material for: Effectiveness and risk of ARB and ACEi among different ethnic groups in England: A reference trial (ONTARGET) emulation analysis using UK Clinical Practice Research Datalink Aurum-linked data
Source: PLoS Med. 2024 Sep 16;21(9):e1004465. doi: 10.1371/journal.pmed.1004465 (PMC11441682; doi:10.1371/journal.pmed.1004465)
Supplement: Attachment — Submitted filename: response_to_reviewers.pdf [file pmed.1004465.s003.pdf]

To the reviews and editors,

Many thanks for your comments which have helped us improve the clarity of our manuscript. We have responded to all comments in the table below, highlighting the changes made. **Page and line numbers provided are from the track changes version.**

Editorial comments	Response
1) We very much appreciate the value in applying trial emulation methods to real-world data. However, we agree with the reviewers and the academic editor that, as written, the manuscript is not without limitations. Specifically, we agree with the concerns raised regarding the use of CPRD Aurum Vs GOLD and with the concerns regarding the potential for selective reporting. We require complete clarification on these points. Please respond in full to all comments detailed below.	Thank you for your comment. Please find responses to all reviewers and editorial comments below.
2) Data Availability<br>Thank you for including a Data Availability Statement (DAS) which requires revision. As the data are not freely available, please describe briefly the ethical, legal, or contractual restriction that prevents you from sharing it. Please also include an appropriate contact (web or email address) for inquiries to the UK CPRD. Please note that this cannot be a study author.	Thank you for highlighting this. The text has now been updated with required details:<br><br>Pg 35, line 922: "No additional data available. Data used in this project cannot be publicly shared due to the terms of our license preclude us from sharing individual patient level data with third parties. Data used in this study was provided from the CPRD under a licence from UK Medicine and Healthcare products Regulatory Agency. Data from CPRD at the UK Medicine and Healthcare products Regulatory Agency is available subject to ethical approval, and can be requested using CPRD's Research Data Governance process (https://www.cprd.com/data-access, all enquiries should be sent to: enquires@cprd.com). Programming code and code lists used in this study are available at: https://github.com/ParisBap/TTE_ONTARGET/."
3) Reporting guidance<br>In view of your use of observational, routinely collected data, we suggest that you report your study according to the STROBE guideline which can be found	We appreciate the suggestion to follow the STROBE guideline. Given that our study uses observational routinely collected health data for pharmacoepidemiology, we have aligned reporting with

here: https://eur01.safelinks.protection.outlook.com/?url=http%3A%2F%2Fwww.equator-network.org%2Freporting-guidelines%2Fstrobe%2F&data=05%7C02%7Cp.baptiste%40qmul.ac.uk%7C0d936feee1d44427ff7b08dc8a41cc42%7C569df091b01340e386eebd9cb9e25814%7C0%7C0%7C638537262409571604%7CUnknown%7CTWFpbGZsb3d8eyJWIjoiMC4wLjAwMDAiLCJQIjoiV2luMzIiLCJBTiI6Ik1haWwiLCJXVCI6Mn0%3D%7C0%7C%7C%7C&sdata=wKdvqfX6x5rNoRd%2Fudvh%2FSr%2BVpCdN1wAwpaYEs7huqI%3D&reserved=0<br>When completing the checklist, please use section and paragraph numbers, rather than page/line numbers, as the latter often change in the event of publication.<br><br>We agree with the academic editor (please see below, point #5) that referring to the PRINCIPLED guidance on emulation studies would be helpful and encourage you to review the guidance.	the RECORD-PE extension to STROBE (https://www.bmj.com/content/363/bmj.k3532).
4) Statistical reporting<br>Throughout, including tables and figures, please quantify the main results with 95% CIs and p values.<br>When reporting p values please report as <0.001 and where higher as p=0.002, for example. If not reporting p values, for the purpose of transparent data reporting, please clearly state the reasons why not. When reporting 95% CIs please separate upper and lower bounds with commas instead of hyphens as the latter can be confused with reporting of negative values.<br>Please include the actual amounts and/or absolute risk(s) of relevant outcomes (including NNT or NNH where appropriate), not just relative risks or correlation coefficients. (example for absolute risks: PMID: 28399126).	Thank you for highlighting this. This has been updated throughout with a sentence added to explain that p values are presented for heterogeneity only.<br><br>Pg 16, line 377: "Corresponding p values for the interaction term in the Cox model and Poisson regression model are presented."
5) Prespecified analysis plan/study protocol<br>Thank you for including your published study protocol as supporting information. If available, please include instead an 'original' version i.e., that would be submitted as part of an ethics/funding approval process.<br><br>For all observational studies, in the manuscript text, please ensure you	Thank you, the original protocol submitted to obtain CPRD data access has now been added. Further detail has been added to the introduction indicating specific hypotheses (pg 8, line 188). We have also added a protocol deviation table to the supplementary material (Table S1).

indicate: (1) the specific hypotheses you intended to test, (2) the analytical methods by which you planned to test them, (3) the analyses you actually performed, and (4) when reported analyses differ from those that were planned, transparent explanations for differences that affect the reliability of the study's results. If a reported analysis was performed based on an interesting but unanticipated pattern in the data, please be clear that the analysis was data-driven.	
6) Abstract layout<br>Please structure your abstract using the PLOS Medicine headings (Background, Methods and Findings, Conclusions).	Thank you, structure has been updated.
7) Author summary<br>At this stage, we ask that you include a short, non-technical Author Summary of your research to make findings accessible to a wide audience that includes both scientists and non-scientists. The authors summary should consist of 2-3 succinct bullet points under each of the following headings:<br>• Why Was This Study Done? Authors should reflect on what was known about the topic before the research was published and why the research was needed.<br>• What Did the Researchers Do and Find? Authors should briefly describe the study design that was used and the study's major findings. Do include the headline numbers from the study, such as the sample size and key findings.<br>• What Do These Findings Mean? Authors should reflect on the new knowledge generated by the research and the implications for practice, research, policy, or public health. Authors should also consider how the interpretation of the study's findings may be affected by the study limitations. In the final bullet point of 'What Do These Findings Mean?', please describe the main limitations of the study in non-technical language.<br>The Author Summary should immediately follow the Abstract in your revised manuscript. This text is subject to editorial change and should be distinct from the scientific abstract. Please see our author guidelines for more information: https://eur01.safelinks.protection.outlook.com/?url=https%3A%2F%2Fjournals.plos.org%2Fplosmedicine%2Fs%2Frevising-your-manuscript%23loc-author-	Thank you for highlighting this. This section has now been added.

summary&data=05%7C02%7Cp.baptiste%40qmul.ac.uk%7C0d936feee1d44427ff7b08dc8a41cc42%7C569df091b01340e386eebd9cb9e25814%7C0%7C0%7C638537262409578383%7CUnknown%7CTWFpbGZsb3d8eyJWIjoiMC4wLjAwMDAiLCJQIjoiV2luMzIiLCJBTiI6Ik1haWwiLCJXVCI6Mn0%3D%7C0%7C%7C%7C&sdata=ez9LO8jBHMPrHuFmYVtp0P4wZ8izXPq3pPjSo6VnixA%3D&reserved=0	
8) Introduction<br>Please ensure to address past research and explain the need for and potential importance of your study. Indicate whether your study is novel and how you determined that. If there has been a systematic review of the evidence related to your study (or you have conducted one), please refer to and reference that review and indicate whether it supports the need for your study.	Thank you. Text has been updated in the introduction to clarify the novelty of this study and highlight the gap in evidence and need for and importance of this study, specifically in relation to the current NICE treatment guidelines.<br><br>Pg 8, line 188: "Recommendations to favour other agents over ACEi in Black patients continue to be debated.[31] Despite evidence that the risk of angioedema is increased among Black populations, little is known about the ACEi-induced angioedema incidence in UK populations. We sought to explore the generalisability of the ONTARGET trial findings among an ethnically diverse population in England using a reference trial emulation approach, which provides an opportunity to study effects in trial-underrepresented groups"<br><br>Pg 9, line 218: "This will provide up to date evidence on whether the UK recommendation to prescribe an ARB in preference to an ACEi is valid, specifically among Black patients receiving these medications in England. To our knowledge, this will be the first study to quantify the treatment effect heterogeneity by ethnicity for the risk of angioedema in a population outside the US."
9) Discussion<br>Please ensure that you present and organize the Discussion as follows: a short, clear summary of the article's findings; what the study adds to existing research and where and why the results may differ from previous research; strengths and limitations of the study; implications and next steps for	Thank you. This has been reorganised, with sub-headings removed and a concise one-paragraph conclusion added.

research, clinical practice, and/or public policy; one-paragraph conclusion. Please avoid the use of sub-headings such that the discussion reads as continuous prose.	
Reviewer 1	
Reviewer #1: Baptiste and colleagues used CPRD Aurum data to determine the effectiveness of ARB and ACEi for selected clinical outcomes and their side effect profile of angioedema in different ethnic groups. This topic is of clinical importance for the diverse UK population.<br>The analysis is statistically sound, and the conclusion is based on the results obtained.<br>It is interesting that changes in systolic blood pressure in ethnic minorities had a much wider overlapping 95% CI than in the white population. Could authors comment on the reason for that? Would it be due to heterogeneity within each ethnic group (Blacks and South Asians), or due to the wider variations in renin-angiotensin system activity in the groups mentioned above?	Thank you for your comment. We anticipate this could be due to multiple reasons. For example, the wider CIs observed in the ethnic minority populations are likely due to smaller numbers of patients contributing to these groups. We agree with your hypothesis that this could also be due to heterogeneity within these ethnic minority groups. A study which explored ethnic differences in reductions in BP observed similar results and showed wider CIs for Black patients compared to non-Black patients (doi:10.1136/bmj.m4080). We have added this point to the discussion (pg 29, line 699).
Reviewer 2 (statistical)	
Thanks for the opportunity to read your manuscript. My role is statistical reviewer, so I have focused on the design, data, and analysis that are presented. I have put general comments first, followed by questions relevant to a specific section of the manuscript (with a page/paragraph reference).<br><br>The manuscript presents an analysis of UK CPRD data, designed to test the effectiveness of ARB vs ACEi in ethnic minority groups, emulating the design of ONTARGET which was an RCT that compared ramipiril to telmisartan for a composite outcome of CV death, MI, stroke, or serious HF. This study uses the outcomes, inclusion criteria, and analysis from ONTARGET, but instead of randomisation uses propensity score matching to compare patients receiving ACE or ARB. Current guidelines recommend either an ACE or ARB as initial treatment for de-novo patients, but there is some evidence that this treatment effect may vary according to ethnicity. Data from UK general practices was linked to hospitalisations and death registry data. The GP data includes records of prescriptions, and self-reported ethnicity. Exposure was	Many thanks for your comments on our manuscript. Please see responses to queries below.

taken from prescription data, with treatment period defined by initiating ACE or ARB, and ending whether there was more than 90 days of treatment gap. Participants with eligible temporal windows of follow-up were used to match ACE/ARB exposure within self-reported ethnicity (white, black, south Asian). Propensity score was developed with a wide range of personal and clinical features. The PS was used as an IPW after trimming extreme weights with the weighted groups achieving a close balance. The main analysis was a Cox model including all participants, with an interaction between treatment effect and ethnicity. Several sensitivity analyses were conducted including a) an on-treatment analysis where follow-up was censored if they switched or changed treatment (like a per-protocol analysis), b) a model with multiple imputation of baseline BP and CR, and c) restricting data to <2011 for Black patients because of a guideline change.<br>The results were similar to ONTARGET, with a very small treatment effect detected in contrast to the original trial. I agree with the authors that the results are in line with trial, and that the differences in nominal significance between the studies is not important. There was no treatment heterogeneity in the main outcome, with heterogeneity detected for CV death and mortality. I agree with the author's conclusion that there are no important differences between the main results and the sensitivity analyses. It looks like by necessity there was some deviations from the original ONTARGET protocol, but I think it would be impossible to completely match using routinely collected data (i.e. particularly around the need to use prescribing data for case selection) and the study is a good match of the original ONTARGET. The analysis for this manuscript is good (a few queries below) and the methods and the results are clear. The well organised supplementary material made the review much easier, thank you.	
The link to the code lists in the attached original protocol paper is broken - and I can't access these from the DOI number either. Could a working link to these be added to the revision?	Thank you for highlighting this. Code lists have now been added to a GitHub repository: https://github.com/ParisBap/TTE_ONTARGET/.

	Pg 35, line 929: "Programming code and code lists used in this study are available at: https://github.com/ParisBap/TTE_ONTARGET/."
Although CPRD data can't be shared, is it possible to share the code used for data management and analysis to an online repository?	Thank you. Programming code has now been added to a GitHub repository, see above.
The results from the Poisson regression model are described as 'on the additive scale'. What link function was used in these models? If a log link was used (which is usually the default) then the interaction will also be on the multiplicative scale.	Thank you for your comment. As you have described, the Poisson model as used to explore heterogeneity on the additive scale by assessing absolute differences in incidence rates. This model was used with a log link, however, to obtain the incidence rates as opposed to incidence rate ratios we subsequently used Stata's *margins* command followed by the *nlcom* command to obtain incidence rate differences for each ethnic groups. As requested, code has now been provided and uploaded to GitHub which further outlines this process.
L9. Paragraph 2. Ethnicity was just self-report from the GP data? The protocol mentions the potential linkage to other datasets to improve key study variables (ethnicity is given as an example), was this possible in the end?	Thank you for highlighting this. Ethnicity was self-reported in both GP data and linked hospital data. To improve completeness as described in (PMID: 24323951), ethnicity was taken from both data sources. We have updated the text to clarify this:<br><br>Pg 12, line 301: "Ethnicity was self-reported in both data primary and secondary care data; those with missing ethnicity were excluded."
P11, Paragraph 4. How was proportional hazards checked? This is important for this study because an important difference or heterogeneity in event rate over time might not be apparent if the treatment effect or treatment effect within a particular ethnicity is not consistent over follow-up time.	Thank you for this comment and for bringing this to our attention. Proportional hazards were checked using a visual inspection of scaled Schoenfeld residuals. We have now also conducted an additional check within strata of ethnicity. We have added this information to the manuscript to clarify this was conducted.<br><br>Pg 15, line 347: "The proportional hazards assumption was assessed using a visual inspection of scaled Schoenfeld residuals plotted against time."

	Pg 16, line 369: "The proportional hazards assumption was again assessed within strata of ethnicity."
P12. Paragraph 3. Could you add more details about the MI model please, e.g. type of method used to generate the imputations, number of imputations.	Thank you. We have now added more information to state that multiple imputation of chained equations was conducted using Stata's *ice* command in Stata MP 17.0. 10 imputations were conducted separately within each ethnic group and each exposure group.<br><br>Pg 17, line 406: "To assess the bias introduced by including variables with missing values in our propensity-score model, we repeated analyses after multiple imputation by chained equations of baseline blood pressure and creatinine, for which we judged it reasonable to assume missing at random. Other variables originally included in the model had <10% missing data so complete cases were included.[42, 43] Values were imputed for baseline variables only, therefore this analysis was assessed for non-kidney related outcomes only. Imputations were generated using Stata's *ice* command, using 10 imputations separately within strata of ethnicity and treatment."
P17, Paragraph 3. Given that 10 components of the primary outcome were tested for heterogeneity of the treatment effect, does multiplicity affect the interpretation about heterogeneity in Death from CV?<br>Similarly - it looks like the angioedema analysis was not initially planned, should the interpretation for this be the same as the main results?	Thank you. We acknowledge that this could affect the results and have added further detail to this limitation in the discussion.<br><br>Pg 30, line 751: "Secondly by repeating all the outcomes of the ONTARGET trial there is a risk of chance findings due to multiple testing, therefore chance should be considered when interpreting the observed heterogeneity."<br><br>We have also highlighted multiple testing as a limitation in the 'What do these findings mean' section (pg 6, line 137). Despite this limitation, there was strong evidence of heterogeneity therefore we think it is still reasonable to highlight this observed heterogeneity in our interpretations.

	Despite angioedema not being a primary outcome, it was a planned safety outcome, as studied in the trial and referenced in the protocol. Due to emulating the design of the ONTARGET trial as closely as possible, which had a specified CV primary outcome we have kept angioedema as a secondary outcome. Due to the importance of this outcome for this population group and the UK treatment guidelines, we think this should be interpreted and considered with equal importance as the primary outcome.
Reviewer 3	
Major: 1- I suppose the authors used propensity scores to estimate the probability of receiving the treatment that the patients indeed received which is better referred to as inverse-probability weighting. In that case, I would use the latter terminology to be clearer.	Thank you, this has now been updated throughout.
Major: 2- The choice of variables in the treatment model is not justified in any detail. Did the authors use a causal graph to identify these factors? What are the potential unmeasured confounders?	Thank you for highlighting this. Variables were chosen based on a priori knowledge and the use of a DAG which has now been added to the supplementary material. Text has been updated to reflect this.<br><br>Pg 14, line 321: "Variables considered as confounders and included in the propensity score model were chosen based on *a-priori* knowledge and the use of a directed-acyclic graph (supplementary Fig S2)."
Major: 3- The on-treatment analysis requires further adjustment for time-varying factors affecting treatment discontinuation which may introduce selection bias due to artificial censoring of the non-adherence individuals. The results of these analyses don't seem to be reported in the text or appendix.	Thank you for this comment. As discussed in literature by Hernan et al., (doi: 10.1056/NEJMsm1605385) it is suggested that adjustment of pre and post baseline factors should be implemented in an on-treatment approach. Results should then be compared to the trial results that adopt a similar approach. Despite having access to the individual patient level data from the trial we did not have access to the outcome data to reanalyse the trial results under this approach. Therefore, since this was not feasible, benchmarking findings from an on-treatment approach which additionally adjusts for post baseline factors against the

	trial which does not consider post-randomisation variables would not be appropriate. However, we recognise this as a limitation and have added the below text to the discussion. Due to the nature of the data source, we also were unable to determine if patients discontinued for clinical reasons so we acknowledge informative censoring may have affected results. However, the number of patients who were additionally censored for discontinuation, switching or dual use was small (2.4%).<br><br>Pg 31, line 761: "We included an on-treatment analysis to emulate the trial per-protocol effect. This analysis additionally censored patients at the end of a trial eligible period, at treatment switching or at start of dual therapy with ARB and ACEi and estimated the on-treatment effect. It is suggested the per-protocol effect should be re-estimated in the ONTARGET trial and emulation adjusting for pre- and post-baseline information that predicts adherence.[30, 48] However, as we did not have access to the outcome data from ONTARGET we were unable to estimate this effect in the RCT. We were also unable to establish whether patients discontinued assigned treatment for clinical reasons, due to the nature of the data source. Therefore informative censoring may have affected results, however, the number of patients who were additionally censored for discontinuation, switching or dual use was small (2.4%)."
Minor: 1- please avoid using 'respectively' and match each estimate with the subgroup/label	Thank you, this has now been updated throughout.
Minor: 2- Abstract: please report NNH for primary outcome in addition to NNT for angioedema and clarify the balance.	Thank you, we have now added the NNH and NNT for outcomes which showed evidence of heterogeneity to the abstract, this includes the outcome of CV-death for Black and White patients.
Minor: 3- Figure 4 (methods and results): please clarify that these estimates are also derived from the weighted population.	Thank you for highlighting this. The footnote has been updated to clarify that the cohort is the inverse-probability—weighted cohort

	used throughout, however the actual BP measurements shown are not weighted.<br><br>“Measurements are taken from the inverse-probability—weighted population cohort, however BP measurements are not weighted.”
Reviewer 4	
Major: A major issue with the manuscript, and analysis, as it stands, is that it uses CPRD Aurum data alone for the analysis. The approved protocol for this study (20_012) indicates that the cohort would initially be selected using CPRD GOLD and only if this was inadequately powered would CPRD Aurum be used - and then in addition to CPRD GOLD. The published protocol (BMJ Open, 2022) states that CPRD GOLD will be used. It is not clear then, why only analyses using CPRD Aurum data have been reported, which raises questions of reporting bias. Either the full analyses should be presented, or a clear explanation given as to why a change has been made from the original protocol.	Thank you. As this work was conducted as part of a PhD project, during the pandemic there were some restraints which meant we had to deviate from the original protocol. As there was a much larger sample size pavailable in CPRD Aurum alone (>37 million eligible for linkage in Aurum vs ~9 million eligible for linkage in GOLD in June 2021 build), combining of the 2 data sources was not required and by using Aurum alone, saved substantial time and resources. For transparency we have decided to include a protocol deviation table to the supplementary material (Table S1) outlining any deviations from the published protocol along with reasons.
Minor: Please note that "Clinical Practice Research Datalink" is the name of the organisation, not the database. Please update the title accordingly.	Thank you, this has been updated accordingly.
Minor: All types of data used should be specified in the title or abstract, including, if word count allows, the full name of the databased used. In this study, it appears that CPRD Aurum, HES Admitted Patient Care, ONS Death Registration data, and the Index of Multiple Deprivation were used. All should be specified in the title or abstract, as well as a clear statement that the study used linked data.	Thank you. We have updated the abstract accordingly and specified in the title that linked data was used.<br><br>Abstract: “We applied a reference trial emulation approach to UK Clinical Practice Research Datalink (CPRD) Aurum data (linked with data from Hospital Episode Statistics, Office for National Statistics and Index of Multiple Deprivation) to study the comparative effectiveness of ARB and ACEi in ethnic minority groups in England, after benchmarking results against the ONTARGET trial.”<br><br>Title: “Effectiveness and risk of ARB and ACEi among different ethnic groups in England: a reference trial (ONTARGET) emulation

	analysis using UK Clinical Practice Research Datalink Aurum-linked data”
Minor: It is not clear from the manuscript as to whether the study population was, as outlined in the approved protocol, limited to patients eligible for linkage to HES APC and ONS Death data. This should be clarified, and - if this was the case - the title and abstract updated to reflect that the study population was limited to England (not UK as a whole).	Thank you. The title and abstract have now been updated to specify England. Additionally, we have added a sentence to the data sources section to clarify that only patients eligible for linkage were included.<br><br>Pg 10, line 250: “Only patients eligible for linkage to these data sources were eligible for inclusion.”
Minor: If any of the codelists or algorithms have been validated (especially for selecting patients and defining the exposure periods) the validation study should be referenced, or details of the methods/results of validation provided.	Thank you. We have added a statement which references some studies which conducted validation of conditions relevant to our study. In addition, despite no formal validation having been conducted on the emulation methods and algorithms used, results from previous related work have now been referenced where these methods have been applied.<br><br>Pg 11, line 287: “The approach follows previously applied methods in which the ONTARGET trial was used to inform design and benchmark findings, before extending to study treatment effects among other underrepresented groups.[39]”<br><br>Pg 10, line 252: “The validity, quality and completeness of diagnoses which are reported in CPRD, including some conditions related to this study have been described previously and showed high positive predictive value for recording in primary and secondary care data.[34-36]”
Minor: The rationale for selecting 90 days as a treatment gap indicating the end of one exposure period and the start of another should be provided. * * How was a random exposure period selected for patients with more than one?	Thank you. We have added further detail to clarify that the choice of 90 days was to allow for repeat prescriptions which is typically observed for antihypertensive medication, as described in the protocol. As described in the ‘Step 3: Balance across exposure groups’ section (pg 14, line 317) a random period was selected for patients with multiple periods. This was conducted using Stata’s *sample* command (code provided)

Minor: Authors should describe the extent to which the investigators had access to the database population used to create the study population. It is assumed that linked data were provided on a study-specific basis by CPRD staff - consideration should be given to including them in the acknowledgements, if not as potential co-authors.	Thank you. This has now been added to the data sources section in addition to recognising the work of CPRD in the acknowledgements<br><br>Pg 10, line 244: "Data were provided by CPRD and NHS England who facilitated individual patient linkage to the additional datasets used."<br><br>Pg 34, line 889: "The authors thank CPRD for providing data for this study. The authors also thank all of the general practices and patients who contributed to the study."
Minor: The authors should confirm that person level linkage was undertaken between the data sources. The methods of linkages and quality evaluation should be referenced.	Thank you. This has now been clearly specified in the data sources section along with a reference provided<br><br>Pg 10, line 244: "Data were provided by CPRD and NHS England who facilitated individual patient linkage to the additional datasets used."
Minor: The selection of the individuals and exposure periods should be described in more detail - potentially by the means of expanding the study flow diagram. This should include any filtering based on data quality, data availability, and/or linkage.	Thank you. Additional information has been added to Figure 1 to provide more detail around filtering.
Minor: It is assumed that several variables had missing data (e.g. blood pressure, BMI, creatinine). The number of patients with missing data should be indicated for each variable of interest, and the methods section should include an explanation of how missing data were addressed.	Thank you for this comment. Supplementary Table S3 (previously S2) contains information on missing data, including proportion of missing and a footnote which explains how missing data were handled. We have made updates to present the number of patients with missing data in addition to percentages and added the information contained in the table footnote to the methods section of the manuscript.<br><br>Pg 17, line 386: "Due to the complexity and resources required to use multiple imputation in an inverse-probability—weighted

	analysis which assesses heterogeneity, and due to most variables having a small amount of missing data, we used a complete records analysis as the main analysis. We assessed potential bias from this approach in sensitivity analyses. Systolic and diastolic blood pressure had 22% missing data and those patients with missing blood pressure at baseline were excluded from analysis. Since these medications are indicated for hypertension in this population, using complete records only is unlikely to bias results. Baseline serum creatinine had 19% missing data and was included with a binary indicator for missing and non-missing and an additional variable where the mean was imputed for missing values. Alcohol consumption was omitted due to missing data >10%, however was considered in the model with a missing value indicator if balance after inverse-probability—weighting was unachieved when omitting this variable.”
Minor: More consideration should be given, in the discussion, to the implications of using data that were not created or collected to answer the specific research question - what about misclassification bias in other variables and/or incomplete capture of drug exposure?	Thank you for this comment. Discussion around information bias has been added to the discussion highlighting the implications of using codes to define exposure, outcomes and covariates.<br><br>Pg 31, line 774: “Read and ICD10 codes were used to assess covariates, exposure status and outcomes in analysis. These are reliant on such codes being recorded by healthcare professionals and could be subject to misclassification. As drugs for similar indications are being compared, the probability of events being misclassified in the exposure is likely to be similar across groups in the study. Therefore, the information bias is likely to be non-differential, and our results obtained would be biased towards the null.”
Minor: Is there any potential for confounding by indication, contraindication or disease severity or selection bias (healthy adherer/sick stopper)?	Thank you. Discussion around potential confounding by indicating has been added to the discussion<br><br>Pg 31, line 771: “Patients who started ARB in CPRD may be healthier than those receiving an ACEi leading to confounding by

	indication, however this is unlikely to differ substantially by ethnicity. Despite these arguments, ACEi appeared to be most effective in reducing blood pressure for White patients when compared with ARB, with no difference observed for Black patients. This could be due differences in sample size between the ethnic groups or due to heterogeneity within ethnic groups. A study which also explored ethnic differences in reductions in blood pressure observed similar results, also showing wider CIs for Black patients compared to non-Black patients.[16] This observation could also be due to confounding by indication, namely sicker patients with uncoded heart failure may be more likely to be prescribed an ACEi. That idea is supported by the increased risk of death associated with ACEi use compared to ARB use among White individuals.”
Minor: The generalisability of the study results should be discussed.	Thank you. We have added some text discussing the generalisability of the trial results to the limitations section of the discussion.<br><br>Pg 30, line 754: “The data sources used may mean our results are only generalisable to patients in England since it is unknown whether similar treatment effects would have been observed for patients in the rest of the UK. Additionally, by applying the ONTARGET trial criteria, results may not be generalisable to the wider English population receiving these medications but instead to a subset who would have met the trial criteria”
Minor: The authors should provide information on how to access the study protocol and programming code.	Thank you. The published protocol is referenced in the methods section (pg 11, line 285). Code lists and programming code have now been added to a GitHub repository: https://github.com/ParisBap/TTE_ONTARGET/ with a link added to the data sharing statement (pg 35, line 929).
Academic editor	
General: This is an interesting study that examines a clinically, and ethically, important question, i.e. are there differences in the effectiveness and side	Thank you for your review. Please find responses to your comments and queries below.

effects of ARB and ACEi in different ethnic groups.<br><br>This research cannot be done using randomised controlled trials, due to limited sample sizes and diversity, so the target trial emulation design is justified and appropriate. The decision to benchmark the emulation against the ONTARGET trial is commendable, since the chances of achieving a perfect benchmark are low! I am therefore not hugely concerned by the relatively trivial differences between the benchmarking trial and the cohort.<br><br>The main problem with the work is a lack of detail on various methodological aspects, which raise concerns about the validity of the work; these need resolving to improve confidence in the study.	
1) I am somewhat concerned that this manuscript is reporting the results of only part of one of the secondary objectives from the protocol. Secondary Objective 2 in the original protocol seeks "To estimate treatment effectiveness and risk in groups underrepresented in trials using EHRs. This will be applied as in secondary objective 1, with a focus on the groups of: black/Asian ethnicity, aged ≥75 years, and females who were underrepresented.". Why then do we only see the results from the ethnicity sub analysis? Unless you read the protocol, it is not obvious that ethnicity is just one of several subgroups being examined. This is important because the more subgroups you examine, the more you are likely to observe treatment effect heterogeneity by chance. It must be made clear that the current study represents just one part of one of the secondary objectives described in the protocol. Even then, without seeing the other sub analyses, for age and sex, I am left wondering if we are just seeing the more 'interesting' results.	Thank you for your comment. You are correct, as stated in the protocol, this work was part of larger project. Due to the importance of these results around ethnicity we have decided to publish this subgroup as a standalone paper. Since submission of this manuscript further results have been accepted in the American Journal of Epidemiology (doi: 10.1093/aje/kwae137) which shares the results from the other subgroups studied. We have now added a sentence to the methods section highlighting this and referencing the results for the other subgroups.<br><br>Pg 11, line 287: "The approach follows previously applied methods in which the ONTARGET trial was used to inform design and benchmark findings, before extending to study treatment effects among other underrepresented groups.[39]"
2) The published protocol in BMJ Open appears to suggest that a matching process will be used to build the CPRD cohort. It reads as follows: "Having obtained individual patient data for ONTARGET participants, we will match patients within the ONTARGET study to the CPRD ACE inhibitor trial eligible exposure period with the closest propensity score for the probability of being included in the trial". Unfortunately, this is not itself clear, and I could not find	Thank you for highlighting this. Whilst we appreciate the editor's comment that a pseudo population with equivalent characteristics to trial participants must be used to conduct a proper benchmark, we believe benchmarking can be conducted in a number of ways depending on the aims and objectives. Since the aim of this work was to extend to underrepresented groups,

a similar process described anywhere in the manuscript. At the moment, it reads like the only weighting that was performed was the propensity score weighting. Table 1 is described as presenting the “baseline characteristics of trial-eligible patients after applying trial criteria included in propensityscore—weighted reference trial emulation analysis compared to ONTARGET”. I read this to mean it reports the demographic characteristics for the weighted population used for benchmarking. In this table, there are some clear differences between the cohort and trial population, such as in the proportion of female participants (which is ~25% in the trial and ~50% in the cohort). To conduct a proper benchmark, a pseudo population should be made from the cohort population that has the same characteristics as the target trial (note that this pseudopopulation need not then be used for the final analyses, since the point is merely to demonstrate that the results would align with the trial when the population mix is the same). It is currently not clear that this has been done. The authors must explain more clearly how they weighted the cohort to match the profile of the target trial.	which requires a more diverse population, this step would merely just demonstrate comparability in a trial-analogous population but would not add any additional validity to the results when extended to underrepresented groups in the trial-eligible population. To support this decision, in previous work accepted in AJE (doi: 10.1093/aje/kwae137), the additional step of matching to trial participants was conducted and did not improve comparability therefore we concluded that propensity score weighting alone was sufficient when emulating this specific RCT. However, we recognise that this may be misleading due to what is presented in the protocol paper, therefore, we have added this deviation to the newly added protocol deviation table in the supplementary material.
3) If, as I suspect, the authors were unable to obtain individual patient data for the ONTARGET trial sample, this needs to be clearly stated as a reason for deviating from the protocol. However, I note that the ethical approval mentions gaining access to this individual patient data.	Please see above. Although access to trial data was obtained, we did not use this to address this study objectives. To avoid confusion, we have removed this from the ethics statement as this is not applicable to this part of analysis.
4) The target estimands should be more explicitly described and Supplementary Table S1 should similarly list the target causal contrasts as a distinct row. Also, Supplementary Table S1 should be a main table, given the importance to the conduct and interpretation of the study.	Thank you, we have added an additional column to the table for the target trial and added rows including causal contrasts and causal effects. We have moved this table to the main manuscript (Table 1).
5) I was disappointed to find that no directed acyclic graph was provided to demonstrate the assumptions of the data generating mechanism and justify the confounding set. Although not essential, DAGs are routinely recommended by guidance on the conduct of target trial emulation studies, such as the ‘Process guide for inferential studies using healthcare data from routine clinical practice to evaluate causal effects of drugs (PRINCIPLED)’. In the absence of a DAG, the authors need to provide an explanation of the process that was used for identifying variables for inclusion in the propensity score model, and some justification for choosing these specific variables.	Thank you, a DAG has now been added to the supplementary material and the following text has been added to the manuscript<br><br>Pg 14, line 321: “Variables considered as confounders and included in the propensity score model were chosen based on *a-priori* knowledge and the use of a directed-acyclic graph (supplementary Fig S2).”

6) In particular, I would like to hear an explanation/justification for the inclusion of the 'eligible period' variables, such as 'year of start of eligible period' and 'time since first eligible period'.	Thank you. To attempt to emulate the trial design, this analysis included prevalent users which in turn meant additional steps were required to address biases. One suggested way to account for these biases and differences in prescribing is a 'prevalent new user design' proposed by Suissa et al. (doi: 10.1002/pds.4107). In this design, which uses matching, eligible periods for the new drug (ARB) would have only been allowed to match to participants in their respective exposure set. Where the exposure set consists of eligible periods from the older drug (ACEi) that started within a specified time period related to the eligible period of interest. As well as this, variables such as time since first prescription would be matched within the exposure set. Despite the encouraging prospects of this design and the potential to address biases, this method can be computationally difficult to implement in large complex data sources. Therefore, after discussions with colleagues who implemented this method in large EHR cohorts and colleagues with experience of attempting to apply this method to ongoing trial emulation analyses, a second approach was developed which attempted to loosely replicate the methodology of the prevalent new user design. Here, similar variables to those that would have aided the exposure set were created. The time-related variables considered included calendar year of start of trial-eligible period, time since first trial-eligible period (irrespective of exposure group), number of prior ARB and ACEi trial-eligible periods prior to the trial-eligible period of interest. These were then included in the propensity score model to account for biases introduced from treatment switching and differences in prescribing when including prevalent users.
7) There are insufficient details on the creation of the propensity score model. As the authors will know, a propensity score approach is only accurate if the correct functional forms are modelled. No details are provided on the how the model was built and how the parametric assumptions were checked. Was linearity assessed for all continuous variables; and how were any non-	Thank you. Further detail has now been added to the supplementary material in appendix 1, in addition to the programming code for building the propensity score model being made available. Since BMI did not have a large amount of missing

linearities addressed? What about non additive / interaction terms? Why was BMI divided into categories (and why weren't height and weight modelled as individual and joint terms)?	data we do not anticipate that by including BMI as opposed to individual height and weight would have impacted results.
8) The authors trim individuals with extreme propensity scores to reduce the risk of positivity violations. It would nevertheless be reassuring to see the propensity score distribution among the two treatment groups, in order to demonstrate good overlap of the two treatment groups across all propensity scores. Please include a supplementary figure showing the propensity score distribution. If there are areas of poor overlap, then additional trimming may be required or at least explored with sensitivity analyses.	Thank you. The distribution plots have now been added to the supplementary material. This is presented for each ethnic group separately as individual propensity score models were used for the three ethnic groups.
9) Since the study is effectively focussed on estimating the effects of ARB and ACEi in specific ethnic groups, the overlap distribution plots should arguably be repeated in each ethnic group.	See above.
10) Insufficient information is provided on the multiple imputation approach. What variables were used for the imputation, above and beyond those included in the propensity score model? What modelling approach was used. Mention of MICE suggests GLMs; was there any exploration of non-linearities? If the missingness model is reasonable, the multiply imputed results should be less biased than the complete cases analyses; why then are these presented in supplementary materials, with the complete case analyses considered the primary analysis?	Thank you. As discussed above, due to the complexity and resources required to use multiple imputation in an inverse-probability—weighted analysis which assesses heterogeneity, and due to most variables having a small amount of missing data, we used a complete records analysis as the main analysis. This approach also prevented us from having to make assumptions about the mechanism of the missing data which could have resulted in further biases. This allowed us to statistically assess heterogeneity, which was the aim of this work, without adding further complexity and assumptions. We have added details on this to the methods section under a missing data section.<br><br>We have also added further details about the multiple imputation approach.<br><br>Pg 17, line 386: "Due to the complexity and resources required to use multiple imputation in an inverse-probability—weighted analysis which assesses heterogeneity, and due to most variables having a small amount of missing data, we used a complete records analysis as the main analysis. We assessed potential bias

	from this approach in sensitivity analyses. Systolic and diastolic blood pressure had 22% missing data and those patients with missing blood pressure at baseline were excluded from analysis. Since these medications are indicated for hypertension in this population, using complete records only is unlikely to bias results. Baseline serum creatinine had 19% missing data and was included with a binary indicator for missing and non-missing and an additional variable where the mean was imputed for missing values. Alcohol consumption was omitted due to missing data >10%, however was considered in the model with a missing value indicator if balance after inverse-probability—weighting was unachieved when omitting this variable."<br><br>Pg 17, line 406: "To assess the bias introduced by including variables with missing values in our propensity-score model, we repeated analyses after multiple imputation by chained equations of baseline blood pressure and creatinine, for which we judged it reasonable to assume missing at random. Other variables originally included in the model had <10% missing data so complete records were included.[42, 43] Values were imputed for baseline variables only, therefore this analysis was assessed for non-kidney related outcomes only. Imputations were generated using Stata's *ice* command, using 10 imputations separately within strata of ethnicity and treatment."
11) I am not clear why the second pre-specified criteria was for the 95% to contain 1, given this was not the point estimate for the ONTARGET trial? Is there some other reason to believe the true effect should be exactly null?	Thank you. Since the ONTARGET trial was a non-inferiority trial which showed comparative treatment effects this criteria was based on these results. At the time of setting this criteria (2019) little work had been done in this area. We decided to base our criteria solely on the ONTARGET trial results, thereby a comparative treatment effect between ARB and ACEi would be consistent with a 95% CI containing 1.

12) The fact that this second pre-specified benchmarking test assumes the perfect null, leads to the study failing to meet this test. Rather than discussing the appropriateness (and over harshness) of this test, the authors instead say that the test wouldn't have been violated if only the sample size had been different! This is a completely disingenuous argument unless all other hypotheses tests are restricted to the same smaller sample size. It is unacceptable to pick and choose your sample size for different tests! In my opinion, the authors have created a rod for their own back by setting an inappropriate benchmarking criteria in their protocol. An honest response to this would be to explain why this test is in fact overly conservative.	Thank you, see above. We recognise that this criteria may be stringent however we ask the reviewers to appreciate that the time the protocol was written these methods were not commonly implemented, therefore little guidance was available on setting criteria. We chose the criteria based on the trial results however we have recognised that it may have been more appropriate to set a criteria that considers sample size which could be useful research implications for future studies. Despite this, we have decided to follow what was set out in the protocol and importantly our study drew comparable results to the trial. We have added a comment to the discussion which highlights the limitations of the benchmarking criteria.<br><br>Pg 30, line 747: "As these methods are more commonly implemented, we anticipate guidance arising in setting benchmarking criteria. Despite our pre-specified criteria being based on the ONTARGET trial results we recognise it may have been more appropriate to consider the implications of increased sample size when setting criteria related to confidence bounds."
