# Supplementary material for: Effectiveness and risk of ARB and ACEi among different ethnic groups in England: A reference trial (ONTARGET) emulation analysis using UK Clinical Practice Research Datalink Aurum-linked data
Source: PLoS Med. 2024 Sep 16;21(9):e1004465. doi: 10.1371/journal.pmed.1004465 (PMC11441682; doi:10.1371/journal.pmed.1004465)
Supplement: Attachment — Submitted filename: Response_to_reviewers_v2.pdf [file pmed.1004465.s004.pdf]

<table>
<tr><td colspan="2">To the reviews and editors,
Many thanks for reviewing our revised manuscript and for your comments and feedback. We have responded to all the remaining comments and queries in the table below highlighted by page and line numbers related to the track changes version of the revised manuscript.</td></tr>
<tr><td>Academic editor</td><td>Response</td></tr>
<tr><td>I have had a look through the response and am very happy with the changes. This is an excellent and important piece of research and I believe it is now sufficiently well reported to be published in Plos Medicine!</td><td>Many thanks for your review of our revised manuscript.</td></tr>
<tr><td>Editor requests</td><td></td></tr>
<tr><td>GENERAL
Thank you very much for your detailed and considered responses to previous editor and reviewer comments. Please see below for further comments which we require you address prior to publication.</td><td>Thank you. Please see responses to comments below.</td></tr>
<tr><td>DATA AVAILABILITY STATEMENT
Thank you for updating your statement. Please remove, ‘No additional data are available.’ From the first line. Please define the abbreviation ‘CPRD’ and please specify Aurum.
Please remove from the main manuscript and include only in the manuscript submission form when you resubmit your manuscript.</td><td>Thank you. This sentence has now been removed from the data availability statement and the statement is now included in the submission form only.

“Data used in this project cannot be publicly shared due to the terms of our license preclude us from sharing individual patient level data with third parties. Clinical Practice Research Datalink (CPRD) Aurum data used in this study was provided from the CPRD under a licence from UK Medicine and Healthcare products Regulatory Agency. Data from CPRD at the UK Medicine and Healthcare products Regulatory Agency is available subject to ethical approval, and can be requested using CPRD’s Research Data Governance process (https://www.cprd.com/data-access, all enquiries should be sent to: enquires@cprd.com). Programming</td></tr>
</table>

	code and code lists used in this study are available at: https://github.com/ParisBap/TTE_ONTARGET/."
AUTHOR SUMMARY<br>Thank you for including an author summary which reads very nicely but in places is a little too long.<br><br>Line 97 – suggest inserting additional bullet point beginning, 'Results suggest…'<br><br>Line 100 – suggest inserting additional bullet point beginning, 'Relative risks…' and consider removing the statistical information to aid brevity and because it may not be accessible to the lay reader.<br><br>Line 108 onwards – this section is pretty lengthy throughout. Suggest revising for brevity in consideration of the below comments.<br><br>Line 112 – sentence beginning, 'Unlike…' could be a separate bullet point, although it does repeat, to some degree, the former sentence so could also be revised/removed. We leave it your discretion.<br><br>Line 119 - suggest moving the sentence beginning, 'Without replication…' and up to '…influence guidelines…' perhaps to the limitation point? And then perhaps combining the sentences either side here?	Thank you for your comments and suggestions. Two additional bullet points (see below) have now been added to the '*What Did the Researchers Do and Find?*' section and the statistical information have been removed (pg 5, line 98 and pg 6, line 102)<br><br>Changes have also been made to the '*What Do These Findings Mean?*' section to make this more concise. The first bullet point (pg 6, line 109) has been updated to: "Unlike traditional RCTs, the reference trial emulation design provides an opportunity to study effects in large, diverse samples with the possibility to identify subgroup effects among trial-underrepresented groups, including South Asian and Black patients". The second bullet point has been condensed and your suggestion to move the sentence beginning, 'Without replication…' to the limitation point has been implemented (pg 6, line 116).
Please remove the competing interest, funding, data availability and include only in the manuscript submission form. The	Thank you, this has been implemented.

transparency and dissemination statements can be removed altogether.	
REFERENCES<br>For in-text reference callouts please place citations in square parentheses separate by commas. For example, [1,3,6] or [1-3]. Please check and amend throughout all sub-sections of the manuscript and supporting files.<br>In the bibliography please ensure that you list up to but no more than 6 author names followed by et al.<br>For all web references please ensure you include an, 'Accessed [date].'<br>Journal name abbreviations should be those listed in the National Center for Biotechnology Information (NCBI) databases.	Thank you. This has been checked and updated where necessary.
SUPPORTING INFORMATION<br>As the supporting information files are contained with a single file as 'S1 Appendix', please apply alphabetical labelling to each table and figure contained within the S1 file. For example, 'Fig A' to 'Fig Z' and 'Table A' to 'Table Z'.<br>Plain text does not need to be labelled and can just be given a title as necessary. For example, 'Statistical Analysis Plan'.<br>Please cite tables/figures as 'Fig A in S1 Appendix and/or 'Table A in S1 Appendix', for example. Please cite plain text as, 'Statistical Analysis Plan in S1 Appendix, for example.<br>Alternatively, supporting information files may be uploaded as separate files and figures/tables can then be labelled as 'S1 Table' (so on) and 'S1 Fig' (and so on). Any additional documents (protocols/analysis plans etc.) can be labelled as 'S1 Protocol', for example. Items should be cited exactly as labelled.	Thank you for highlighting this. In text citations and labels have been updated with alphabetical labelling.<br><br>The RECORD-PE checklist has been updated to include section and paragraph numbers related to the latest version of the manuscript.

Either approach is fine but not a combination as it disrupts accurate hyperlinking of items at the time of publication.<br><br>RECORD-PE checklist – thank you for including this. Please amend the checklist referring to section and paragraph numbers as opposed to page numbers as the latter often change at publication.	
SOCIAL MEDIA<br>To help us extend the reach of your research, please detail any X (formerly Twitter) handles you wish to be included when we tweet this paper (including your own, your coauthors', your institution, funder, or lab) in the manuscript submission form when you re-submit the manuscript.	Thank you, this has been added.
Reviewer 2	
Thanks for the revised manuscript and responses to my original review. The updated manuscript resolves all my original questions. All the readily available study materials (ICD10 codes and syntax) are now available on a repository.<br><br>One of the reviewers noted that the original protocol planned to use CPRD GOLD, but instead used CPRD Aurum. The authors have a reasonable explanation for this and have put this in an appendix. In my opinion, using a larger database to save time and money (particularly if there was a PhD student dependent on accessing the data) is a reasonable excuse to change from the protocol.	Thank you for your review.
Reviewer 4	
Thank you for responding to my previous comments, and those of fellow reviewers - the paper is much improved and my remaining comments are minor only.	Thank you for your comments. To enable us to emulate the design of a trial, whereby patients could meet the trial criteria on more than one occasion, we considered multiple exposed periods and

Whilst it is positive that a treatment gap was included to allow for repeat prescriptions, it is not clear why this needed or why 90 days (as opposed to any other length) was needed. Please expand.	assessed the trial criteria at each of these points. Due to the nature of the data source, we considered continuous courses of therapy as exposed periods. To allow us to consider multiple exposed periods it was required to separate patients' treatment history into exposed and unexposed periods. We assumed that when a treatment gap had occurred the exposed period had ended and a potential new exposed period could begin that would be considered in analysis. As patients are likely to receive these medications on repeat prescriptions we chose 90 days as opposed to 30 days to allow for this case, therefore 90 days was the minimum gap we could confidently assume a patient had been unexposed had they not received a prescription during this time. Using this minimum treatment gap as opposed to a longer time point such as 180 days allowed us to consider a patients eligibility at the maximum number of time points.
The additional information added to Figure 1 is appreciated - however, it would be of benefit to start the figure with the number of patients overall (eligible or ineligible for linkage) prior to restricting to those eligible for linkage.	Thank you. Unfortunately, due to the space limitations and the size of this cohort, data was restricted to those who were eligible for linkage therefore the number of patients with a prescription within the study period (regardless of linkage eligibility) is not easily accessible. I could request this count from CPRD however it is likely to take some time to receive this. We have however added the number of patients in Aurum prior to linkage to Figure 1 which shows the proportion of patients who are lost due to not being eligible for linkage.
The additional discussion of information bias is beneficial - but it would be worth reiterating that CPRD data would not include prescriptions given outside of primary care, and to consider whether misclassification of exposure is still anticipated to be non-differential in this case.	Thank you for highlighting this. This limitation bas been added to the discussion (pg 31, line 647):<br><br>"Read and ICD10 codes were used to assess covariates, exposure status and outcomes in analysis, with prescriptions only being recorded in primary care. … However if there are differing rates of

	prescribing between ACEi and ARB is secondary care settings for this population group, the bias could be differential.”
